# Immunization-induced antigen archiving enhances local memory CD8+ T cell responses following an unrelated viral infection

**DOI:** 10.1038/s41541-024-00856-6

**Published:** 2024-03-21

**Authors:** Thu A. Doan, Tadg S. Forward, Johnathon B. Schafer, Erin D. Lucas, Ira Fleming, Aspen Uecker-Martin, Edgardo Ayala, Jenna J. Guthmiller, Jay R. Hesselberth, Thomas E. Morrison, Beth A. Jirón Tamburini

**Affiliations:** 1 Department of Medicine, Division of Gastroenterology and Hepatology, University of Colorado School of Medicine, Aurora, CO USA; 2 Immunology Graduate Program, University of Colorado School of Medicine, Aurora, CO USA; 3 Medical Scientist Training Program, University of Colorado School of Medicine, Aurora, CO USA; 4 Department of Biochemistry and Molecular Genetics, University of Colorado School of Medicine, Aurora, CO USA; 5 Department of Immunology and Microbiology, University of Colorado School of Medicine, Aurora, CO USA

**Keywords:** Listeria, Protein vaccines

## Abstract

Antigens from viruses or immunizations can persist or are archived in lymph node stromal cells such as lymphatic endothelial cells (LEC) and fibroblastic reticular cells (FRC). Here, we find that, during the time frame of antigen archiving, LEC apoptosis caused by a second, but unrelated, innate immune stimulus such as vaccina viral infection or CpG DNA administration resulted in cross-presentation of archived antigens and boosted memory CD8 + T cells specific to the archived antigen. In contrast to ”bystander” activation associated with unrelated infections, the memory CD8 + T cells specific to the archived antigen from the immunization were significantly higher than memory CD8 + T cells of a different antigen specificity. Finally, the boosted memory CD8 + T cells resulted in increased protection against *Listeria monocytogenes* expressing the antigen from the immunization, but only for the duration that the antigen was archived. These findings outline an important mechanism by which lymph node stromal cell archived antigens, in addition to bystander activation, can augment memory CD8 + T cell responses during repeated inflammatory insults.

## Introduction

Many currently available vaccines can elicit neutralizing antibodies with the primary outcome of vaccine immunogenicity being assessed through surrogate markers such as antibody titers^1–3^. However, antibody neutralization relies on the recognition of surface-exposed epitopes that are highly mutagenic and many pathogens can escape pre-existing antibody-mediated immunity. Specifically, rapidly mutating pathogens such as coronaviruses, HIV and influenza viruses can evade the humoral immune responses that most vaccines generate^4–6^. However, the long-lasting T cell population and its diverse TCR repertoire recognize a small number of immunodominant peptides associated with numerous virus-encoded amino acid sequences that have MHC binding motifs^7–9^. In addition to humoral responses, T cell responses are critical to induce the most efficacious protection against pathogens. In SARS-CoV-2 infections, antibodies produced during the early phase of infection decline over time^10–14^ as seen in a cohort of SARS-CoV-2 convalescent patient IgG responses, which waned after 6 months, while T cell responses were stable for up to 1 year^15–17^. Thus, T cells produce durable protective responses following vaccination resulting in viral clearance of SARS-CoV-2^18,19^. Furthermore, mRNA-lipid nanoparticle (LNP) vaccines elicit both antibody and T cell-mediated responses that work synergistically to provide immunity against SARS-CoV-2 and impede disease progression^16,20^. Therefore, understanding factors that influence how T cell-mediated immunity is generated and re-called is critical to improving current vaccine regimens.

Many studies have established that viral-derived antigens persist for extended periods of time within lymph nodes following viral infection^21–25^. These findings have important implications for the development of vaccines and immunotherapies as they suggest that encouraging antigen persistence may be an effective strategy for boosting immune responses to viral infection. For example, influenza virus antigens that persist can recruit memory T cells, and provide protection against reinfection^21–23,25–27^, which suggests that persisting antigens play a critical role in augmenting memory T cell responses to viral infections. Recently, we demonstrated that a subunit immunization consisting of either a TLR agonist (polyI:C) with an agonistic anti-CD40 antibody or a conjugated TLR-antigen caused the persistence of the antigen in the draining lymph node^28–30^. Similar to virus-derived antigen, persisting antigen from immunization also improves T cell memory^28^. This type of antigen persistence, which we termed “antigen archiving”^28^, is mediated by lymph node stromal cells and differs from chronic viral infections seen in patients with human immunodeficiency virus (HIV) or mouse models of lymphocytic choriomeningitis virus (LCMV) where lack of resolution of the infection leads to T cell exhaustion and eventual immune dysfunction due to chronic engagement of the adaptive immune response^31–34^. Antigen archiving was instead defined as the induction of an active immune response that facilitates the increased duration of antigen within lymph node stromal cells^28–30,35,36^. Antigen archiving was not dependent on the type of antigen administered^28^, although antigen size-based on the ability of the antigen to traffic through the lymphatics^37^ was a contributing factor. Furthermore, conjugation of a TLR agonist to a protein antigen increased the duration of antigen in DCs and possibly LECs or fibroblasts^30,38,39^.

Lymph node stromal cells are comprised of three main subsets, which include fibroblastic reticular cells (FRC), lymphatic endothelial cells (LEC), and blood endothelial cells (BEC) - each of which can be subsetted further based on transcriptional profiling^30,40–44^. Different lymph node stromal cells are capable of retaining antigens. Follicular dendritic cells, a fibroblast subset, acquire antibody:antigen immune complexes, which are held in non-degradative endosomal compartments that can be recycled to the surface for antigen sampling by B cells^45^. Follicular dendritic cells hold multiple different types of immune complexes in their recycling endosomes, allowing for a diverse range of antigens to be presented to B cells^45–47^, resulting in the generation of robust plasma cell responses and high levels of specific antibodies that can neutralize antigens^46^. Our previous work demonstrated that LECs have the capacity to store protein antigens following immunization and viral-associated antigens for prolonged periods of time and that antigens retained by LECs are important for T cell protective immunity^28–30^. By labeling antigen with nucleic acid or fluorescent tags prior to immunization, we detected antigen in the lymph node by single-cell RNA sequencing and flow cytometry predominantly in LEC subsets including floor, ceiling, collecting, and Ptx3 LECs at 2–5 weeks post-vaccination^28,30^. Although LEC presentation of self-antigens^48,49^ or non-adjuvanted antigens^50^ is tolerogenic, we demonstrated that archived antigens are not presented by LECs directly to CD8 + T cells, but rather are transferred from LECs to migratory conventional DCs (cDCs)^28,29^. The specific mechanism by which antigen exchange occurs between LECs and DCs is unclear but some potential mechanisms we identified include cell-cell interactions between migratory DCs and antigen-bearing LECs, endocytosis of apoptotic LECs by the DCs^29^, or possibly through capture of exosomes secreted by the LECs. Upon acquisition of antigens from LECs, migratory cDCs process and present antigenic peptides by MHC class I to memory CD8 + T cells^29^. Adoptive transfer of T cell receptor transgenic CD8+ T cells indicate that presentation of archived antigen to memory CD8 + T cells even at late time points after vaccination increases the number of antigen-specific memory CD8 + T cells with enhanced cytotoxic capabilities during an antigenic re-challenge with *Listeria monocytogenes (LM)-*expressing ovalbumin (ova)^28^. Consequently, mice challenged with LM-ova had a lower bacterial burden and thus enhanced protection against infection^28^. Thus, LEC antigen archiving is an important process by which DCs acquire foreign antigens at late time points post-immunization or viral infection to enhance protective immunity.

While antigen archiving appeared to improve protective memory responses through the slow release of antigens during lymph node contraction^29^ it was still unclear if archived antigens could benefit protective immunity during another inflammatory event that resulted in LEC expansion and contraction. Indeed, others have demonstrated that memory CD8 + T cells can be stimulated as a result of heterologous immunity^7^. At least one of the mechanisms by which heterologous immunity is conferred is through ”bystander” activation. Bystander activation occurs as a result of cytokine (e.g. IFNα, IL18, IL15) produced during viral infection, but independent of antigen recognition by the T cell receptor^51^. Bystander activation can lead to improved protection against heterologous challenge via increased production of IFNγ by non-specific T cells^52,53^. Based on the capacity of memory CD8 + T cells to respond more readily than naïve CD8+ T cells to lower levels of antigen and cytokines in the microenvironment^51,54,55^, this phenomenon is unsurprisingly driven by memory T cells. Whether archived antigen is an additional mechanism by which memory CD8 + T cells can be stimulated more specifically to push them into a secondary or tertiary memory state with an increased capacity to proliferate and produce cytokines^56,57^ is unknown.

Unresolved questions regarding the prior work include whether an unrelated inflammatory stimulus can promote increased antigen-specific memory T cell protective responses as a result of archived antigen, and whether the benefits of antigen archiving are local or systemic. Here, we explore how LEC handling of archived antigens during an unrelated infection impacts the immunization-induced memory T cell responses and protection. We found that once the vaccine antigens were archived, a secondary VV-WR infection or CpG DNA injection caused a significant increase in archived antigen-specific memory CD8 + T cells. To this end, we confirmed that an unrelated innate immune stimulus caused both LEC proliferation (3–6 days) and apoptosis (2–3 weeks)^28,29,36,58^. This observed increase in antigen-specific CD8 + T cells was partly due to cytokine-induced “bystander activation”, but also a result of T cell receptor (TCR) engagement (antigen specific). Interestingly, enhanced protection to ovalbumin or SARS-CoV2 receptor binding domain (RBD) administered in the immunization (polyI:C/αCD40) was only observed locally. Taken together, our data demonstrate that LEC-archived antigens, such as ovalbumin or RBD, impact downstream memory CD8 + T cell responses during an unrelated infection and identify a mechanism that leads to superior CD8 + T cell effector function during an antigenic rechallenge.

## Results

### Lymphatic endothelial cells archive antigens following immunization

**Fig. 1:**
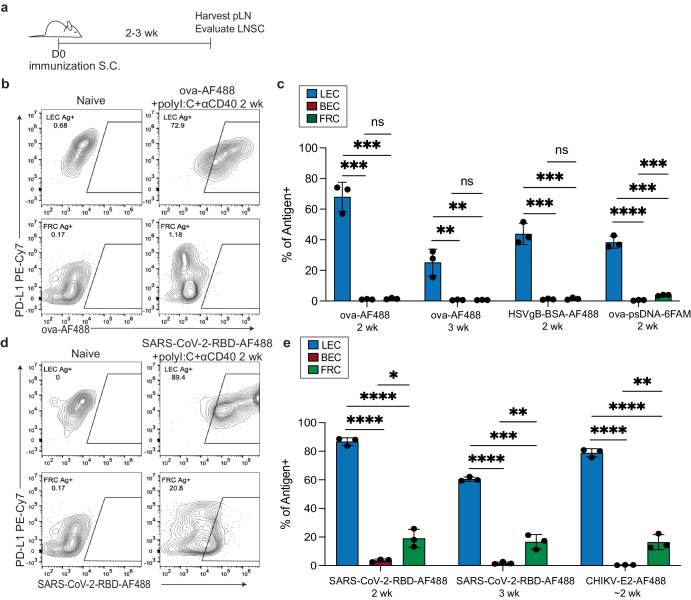
Antigen levels in lymph node stromal cells after immunization. **a** Experimental schematic for **b**–**e**. C57/BL6 mice were immunized subcutaneously in the footpad and/or flank with the indicated antigens and adjuvants. **b** Cells were stained with CD45, PDPN, CD31 and PD-L1. Cells were gated on CD45-PDPN + CD31- for FRC and CD45-PDPN + CD31+ for LECs. Shown are examples of LEC and FRC antigen-positive cells based on PD-L1 expression (floor, MARCO LEC) and ova-AF488+ from mice 2–3 weeks after immunization with ova conjugated to Alexa-Fluor 488 (AF488) and polyI:C and αCD40. **c** Quantification of the frequency of LEC, BEC, and FRC in the popliteal lymph node (pLN) that are positive for the indicated antigens administered with polyI:C and αCD40 at indicated time. **d** Same as (**b**) except for mice were immunized with SARS-CoV-2-RBD-AF488, polyI:C, and αCD40. **e** Same as in (**c**) except for SARS-CoV-2-RBD and CHIKV-E2 with polyI:C and αCD40. CHIKV-E2 was repeated for 9–14 days post-vaccine (~2 weeks). Statistical analysis was done using an unpaired *t*-test where the *p*-value between naïve and indicated antigen is <0.0001. In each experiment, at least *n* = 2–3 mice per group were evaluated and the experiment was repeated *n* = 2–5 times for **c**–**e**. Shown is the representative data from one of the experiments. Error bars are mean ± standard error of the mean. ns not significant, *p < 0.05, **p < 0.01, ***p < 0.001, ****p < 0.0001.

We previously discovered that LECs store soluble ovalbumin (ova) antigen both at the single-cell level and within whole lymph node tissue by using conjugated DNA tags as well as fluorescent tags that label the antigen^16,20,21^. Here, we further build on these previous findings by showing that various protein antigens are archived for 2–3 weeks by LECs in the draining lymph node after subcutaneous immunization (Fig. 1a). By gating on CD45- cells we were able to discern the three main lymph node stromal cell populations: LECs, FRCs, and BECs based on the expression of podoplanin (PDPN) and CD31 (Fig. 1a and Supplementary Fig. 1a). To better visualize different LEC subsets we also stained cells with anti-PD-L1 which is expressed by floor and Marco+ LECs^44^. Using a number of different types of antigens and TLR agonists, we assessed antigen localization at 2–3 weeks post-immunization. In the presence of a combination adjuvant that includes polyI:C, a TLR3 agonist, and an agonistic anti-CD40 antibody (αCD40), we confirm that LECs archive fluorescently labeled ova (Fig. 1b, c). Moreover, this observed phenomenon is not specific to ova protein as we also found that HSV-derived SSIEFARL peptide conjugated to bovine serum albumin (BSA) (HSV-gB-BSA-AF488) accumulates in LECs (Fig. 1c). To further address whether this observation was specifically polyI:C-dependent, we immunized mice with ova conjugated to phosphorothioated DNA (ova-psDNA), which engages TLR9, and observed comparable levels of ova positive LECs to polyI:C (Fig. 1c). To assess whether different protein antigens also accumulate in LECs or other cell types, we evaluated the SARS-CoV-2 receptor binding domain (RBD) protein and the chikungunya virus E2 glycoprotein (CHIKV-E2) (Fig. 1d, e), both administered in combination with polyI:C and αCD40. We found that the SARS-CoV2-RBD was also acquired and archived by LECs after immunization, but in contrast to albumin-based antigens was also acquired by FRCs to a lesser degree (Fig. 1d, e). Interestingly, within the FRC population, the RBD protein levels were maintained from 2 weeks to 3 weeks (Fig. 1d, e), however, levels were only about one third of the amount acquired by LECs. Additionally, SARS-CoV-2-RBD was present in both PD-L1^hi and low^ LEC populations, but the fluorescence intensity was increased in the PD-L1^hi^ floor/MARCO LEC populations (Fig. 1d). Finally, when evaluating recombinant CHIKV-E2 we noticed that again, both LEC and a small frequency of FRCs acquired the E2 protein at ~2 weeks post-vaccination. Of note, CHIKV E2 is the required protein necessary for viral entry into LEC and FRC populations via the receptors MARCO^41^ and MXRA8^59,60^, respectively. Similar to CHIKV infection there was more detectable E2 within the LEC than FRC populations^41^. There was minimal detection of antigens in BECs (Fig. 1c, e). To confirm antigen was functionally archived, we utilized TCR transgenic T cells specific for ova or HSV-gB-BSA. Ova is presented to OT1 TCR transgenic T cells, recognizing the dominant ova epitope—SIINFEKL, while the BSA-SSIEFARL is presented to gBT, recognizing the SSIEFARL epitope^61,62^. We transferred carboxyfluorescein succinimidyl ester (CFSE)- or violet proliferation dye (VPD)-labeled TCR transgenic T cells into mice at 2–3 weeks post-immunization (Supplementary Fig. 1b–e). Three days after T cell transfer, T cell proliferation in the draining lymph node was assessed by CFSE or VPD dilution (Supplementary Fig. 1b–e). Both OT1 and gBT T cells responded to their cognate antigen, demonstrating the presence of archived antigens within the host 2–3 weeks post-vaccination that can be presented to T cells. These data confirm that lymph node stromal cells archive a wide array of antigens during an active immune response and that there may be some cell type specificity based on the type of antigen delivered.

### LEC apoptosis following an unrelated infection coincides with cDC1 archived antigen presentation

**Fig. 2:**
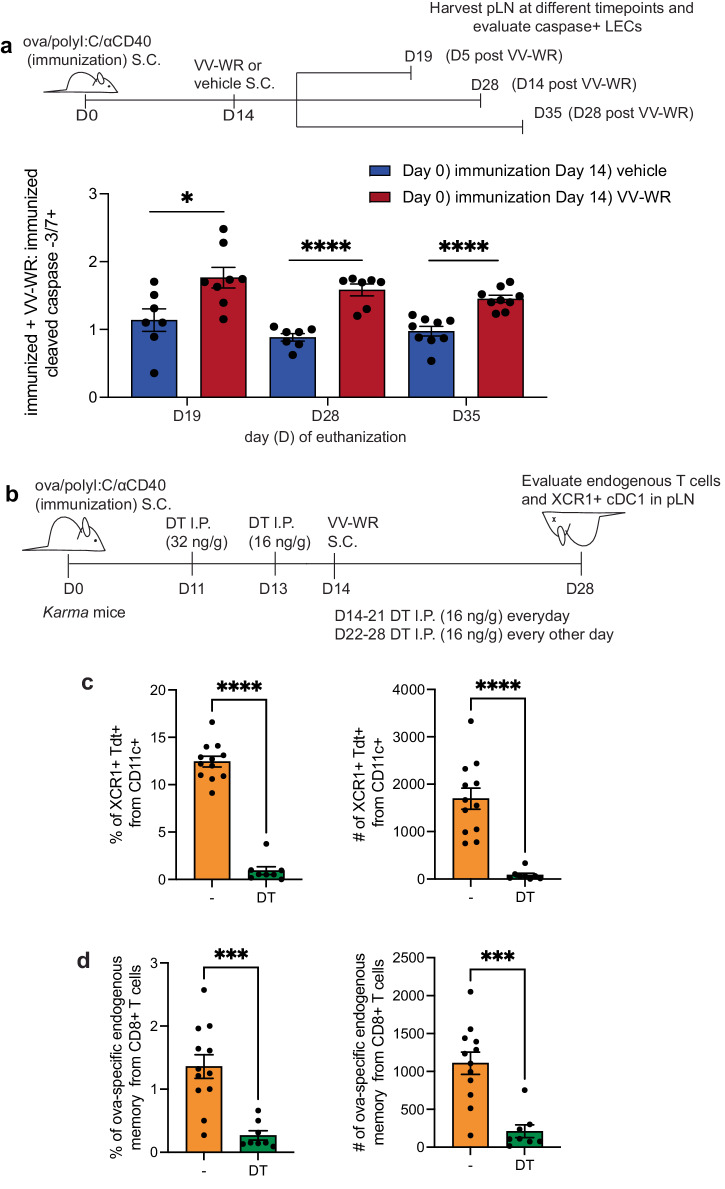
Increased LEC apoptosis following VV-WR coincides with cDC1 presentation of archived antigens. **a** Experimental schematic. Mice were immunized and infected with VV-WR at indicated time points. Popliteal lymph node (pLN) were harvested at respective time points and cleaved caspase 3/7+ LECs were evaluated. Quantification of the fold change of each treatment at the respective timepoint over vaccine only. The fold change was calculated by taking the percentage of cleaved caspase-3/7 + LEC and dividing by the percentage of cleaved caspase-3/7 + LEC for immunization only at each time point. In each experiment, *n* = 2–5 mice per group were evaluated and the experiment was repeated *n* = 2–3 times depending on the time-point. Shown is the combined data from all experiments. **b** Experimental schematic for (**c**, **d**) *Karma* mice were immunized and infected with VV-WR at indicated timepoints as in a. Except, *Karma* mice were treated at indicated timepoints with DT as described in figure and methods. **c** Quantification of flow cytometric analysis, performed on day 28 after euthanization (CO_2_ followed by cervical dislocation), of cDC1s (CD11c^hi^MHCII^hi^ XCR1 + CD11b-) in vehicle (orange) or DT depleted (green) mice. Shown are percent and numbers from draining popliteal lymph node. **d** As in (**c**) except cells were gated as CD44^hi^ and SIINFEKL tetramer + (ova specific) from (B220 - CD8 + lymphocytes). Shown are percent and number from draining popliteal lymph node. In each experiment *n* = 3–5 mice per group were evaluated and the experiment was repeated (*n* = 3). Shown is combined data from three independent experiments. Error bars are mean ± standard error of the mean. Statistical analysis was done using an unpaired *t*-test where the *p*-value is indicated by an asterisk. ns = not significant, *p < 0.05, ***p < 0.001, ****p < 0.0001.

As we were interested in how LECs impact the downstream immune response, and based on our findings that ova is archived specifically by LECs, all remaining studies were performed with ova as the archived antigen. In response to VV-WR infection, we confirmed with caspase 3/7+ staining that LECs undergo increased apoptosis during lymph node remodeling post VV-WR infection (Fig. 2a, Supplementary Fig. 2a–e). Our previous studies demonstrated that LEC apoptosis is one mechanism by which archived antigens can be acquired by migratory cDCs^29^. Cross-presentation by MHC-class I of cell-associated antigens is performed by conventional dendritic cells type-1 (cDC1) that express XCR1^63^. cDC1 cross-presentation of cell-associated antigens is, at least in part, a result of their capacity to internalize apoptotic cells or cell debris^64–66^. Based on this we asked whether, at 2 weeks post VV-WR infection, when there are high numbers of apoptotic LECs, cDC1s could cross-present LEC archived antigens from the immunization administered 1 month prior. To evaluate this we used *Karma* mice where the fluorescent tandem dimer Tomato (tdtomato) and the human diptheria toxin receptor (DTR) were knocked into the *a530099j19rik* gene and allow for tracking and depletion of XCR1 + cDC1s^67^. We first immunized *Karma* mice with ova/polyI:C/αCD40 subcutaneously in the footpads. Starting 11 days later we administered diptheria toxin every day, or every other day, intraperitoneally until day 28 when mice were euthanized (CO_2_ followed by cervical dislocation). At day 14 mice were injected subcutaneously in the same location as the ovalbumin immunization, but this time with VV-WR, a strain of vaccinia virus that contains no ova-derived epitopes (Fig. 2b). Using this methodology were were able to sufficiently deplete the XCR1 + cDC1s from the lymph node draining the immunization and infection (Fig. 2c, Supplementary Fig. 2f, g). We found that loss of XCR1 + cDC1s resulted in a significant reduction in the ova specific CD8+ T cell population observed 1 month after ova/polyI:C/αCD40 when VV-WR was administered 2 weeks after immunization to induce apoptosis (Fig. 2d, Supplementary Fig. 2h, i). Together, these findings suggest that, during LEC apoptosis caused by an unrelated inflammatory event, LEC associated archived antigens are acquired by cDC1s and cross-presented to CD8 + T cells.

### Endogenous antigen-specific memory CD8 + T cells accumulate following vaccinia infection

**Fig. 3:**
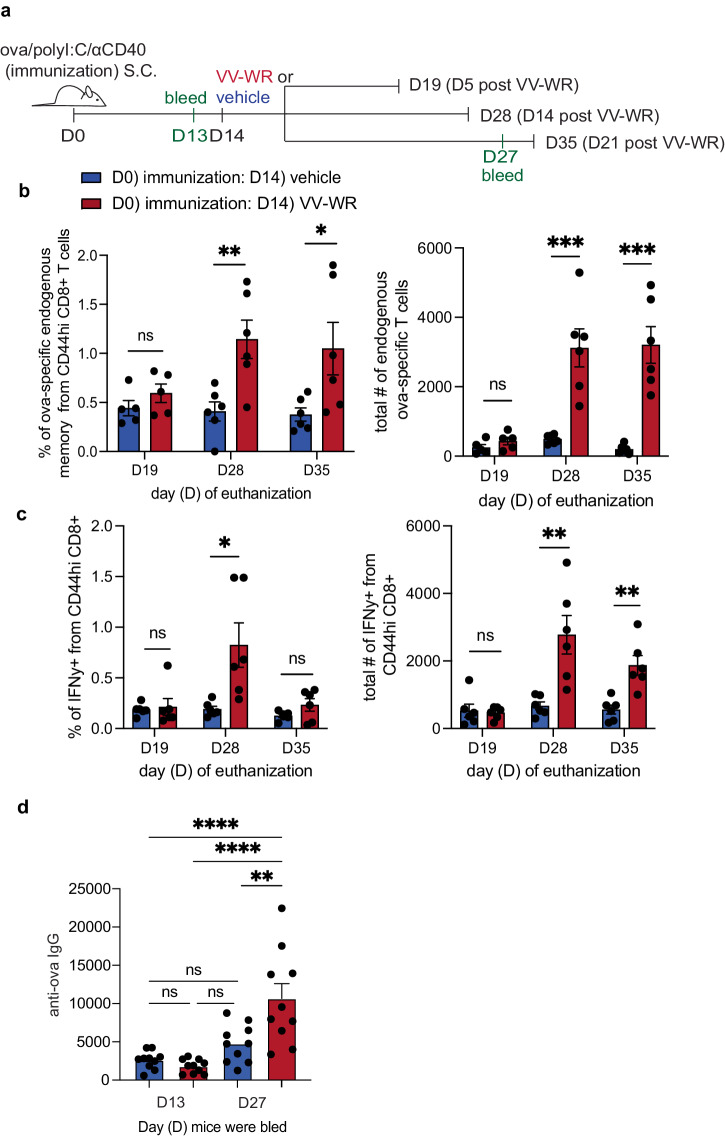
Increased immunization-specific memory CD8 + T cell quantity and function following vaccinia infection. **a** Experimental schematic for (**b**–**d**). Mice were immunized subcutaneously in the footpad with a subunit immunization containing ova, polyI:C, and αCD40. Two weeks later, mice were infected with VV-WR or vehicle (PBS). At time of euthanasia (CO_2_ followed by cervical dislocation) popliteal lymph nodes (pLN) were harvested at respective time points post-VV-WR infection. Half the cells were used to evaluate endogenous CD8 + T cells and the other half were used for ex vivo stimulation with SIINFEKL peptide. **b** Quantification of frequency and number of ova-specific endogenous memory CD8 + T cells in the draining popliteal LN. In each experiment, *n* = 2–5 mice per group were evaluated and each experiment was repeated 3–7 times depending on the time point with similar results. Shown is the representative data from two of the experiments. **c** Quantification of frequency and number of IFNγ -producing B220-CD44^hi^ CD8 + T cells from the draining popliteal lymph node. In each experiment, *n* = 2–5 mice per group were evaluated and each experiment was repeated 3–7 times depending on the time point with similar results. Shown is the representative data from two experiments. (**d**) Quantification of IgG specific antibodies to ovalbumin in the serum using ELISA after the indicated times/treatments from (**a**). Shown are combined data from two independent experiments with *n* = 5 mice per group. Statistical analysis was done using an unpaired *t*-test where the *p*-value between vaccine + vehicle (blue bar) and vaccine + VV-WR (red bar) is <0.0001. Error bars are mean ± standard error of the mean. ns not significant, *p < 0.05, **p < 0.01, ***p < 0.001, ****p < 0.0001.

We next asked if LEC apoptosis following an unrelated viral infection, during the time frame of antigen archiving, impacted the phenotype and/or function of memory CD8 + T cells in vivo. To answer this question, mice were immunized with a subunit vaccine containing ovalbumin, polyI:C, and αCD40 to establish archiving of ovalbumin. Fourteen days later, mice were infected with VV-WR to evaluate the frequency and function of ova-specific CD8 + T cells at 5, 14, or 21 days post-VV-WR infection (Fig. 3a). As these time points reflect the phases of LEC and lymph node expansion and contraction post-VV-WR infection as well as the amount of VV in the lymph node (Supplementary Fig. 2 and Fig. 2a), we could further establish a time frame by which ova-specific CD8 + T cells expanded and responded to an unrelated infection. At 5 days post-VV-WR infection, there was no significant increase in the number of ova-specific CD8 + T cells within the draining popliteal lymph node compared to mice that were injected with the vehicle control (Fig. 3b, Supplementary Fig. 3). However, at 14 and 21 days post-VV-WR infection, endogenous ova-specific CD8 + T cells accumulated within the draining lymph node at a significantly higher degree compared to vehicle-injected mice (Fig. 3b, Supplementary Fig. 3c). Moreover, these T cells were functionally enhanced in their ability to produce IFNγ after ex vivo stimulation with SIINFEKL peptide (an ova-derived epitope) (Fig. 3c, Supplementary Fig. 3d). We found that the IFNγ response by ova-specific CD8 + T cells isolated from the VV-WR-infected mice was significantly higher than the uninfected mice even though neither group was challenged with the ova antigen after initial ova/polyI:C/αCD40 subunit immunization (Fig. 3C). Furthermore, when using RBD as the immunization antigen with polyI:C and αCD40 prior to VV-WR we found increased IFNγ production following ex vivo stimulation with VVLSFELL peptide (an RBD-derived epitope SPIKE_511-518_)^68^ (Supplementary Fig. 4), which suggests the increased cytokine production is not ovalbumin dependent. Finally, to assess differences in other adaptive immune cells we evaluated CD4+ T cell activation in the lymph node (Supplementary Fig. 3e) and circulating anti-ovalbumin immunoglobulin type G (IgG) responses in the serum (Fig. 3d). We found both increased activation of CD4 T cells in the lymph node and elevated ovalbumin specific IgG levels in the serum after VV-WR injection suggesting increased accessibility to antigen and/or bystander activation of multiple cell types. To determine if this increased responsiveness to archived antigen by CD8 + T cells was a result of the potent pro-inflammatory environment caused by VV-WR infection we asked if a non-infectious inflammatory stimulus could produce the same result. We again immunized mice with ova/polyI:C/αCD40 and 2 weeks later administered CpG, a TLR9 agonist, as the secondary inflammatory stimulus in lieu of VV-WR (Supplementary Fig. 5). As with VV-WR infection, we found a significant increase in the number of ova-specific memory CD8 + T cells following local administration of CpG that was dependent on TLR9 (Supplementary Fig. 5). Together, these data suggest that cDC1s, known for their role in presenting apoptotic cell associated antigens, cross-present archived antigens during the time frame of LEC contraction. In turn, this results in the expansion of endogenous memory archived antigen-specific CD8+ T cells following VV-WR infection or CpG DNA injection.

### Non-archived antigen-specific memory CD8 + T cells are stimulated in the absence of antigen after vaccinia infection to a lesser degree than archived antigen-specific memory CD8 + T cells

**Fig. 4:**
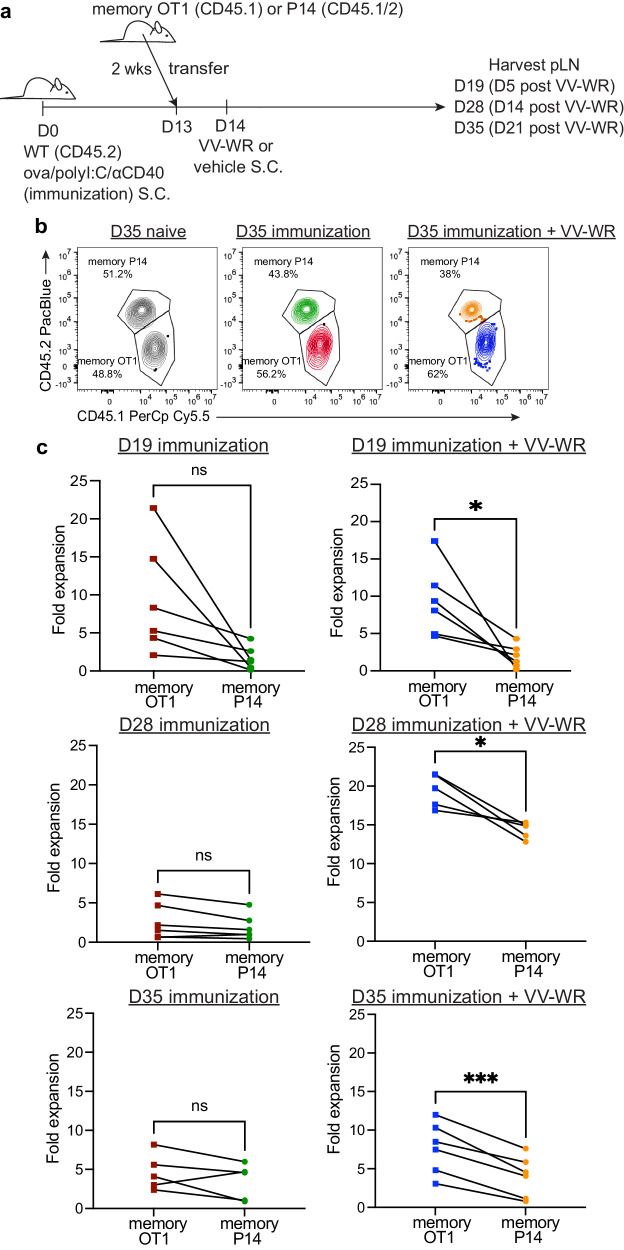
Archived antigen-specific memory CD8 + T cells derived from immunization expand preferentially following vaccinia infection. **a** Experimental schematic for (**b**, **c**). Mice were immunized and infected with VV-WR as in Fig. 2. One day prior to VV-WR infection congenically different memory OT1 and memory P14 CD8 + T cells were isolated following euthanasia (CO_2_ followed by cervical dislocation) and transferred intravenously into WT mice. To establish memory, naïve OT1 or P14 cells were transferred into naïve WT mice and immunized with their cognate antigen (ovalbumin or gp33 peptide) and isolated by CD8 negative selection 2–6 weeks later as described in materials and methods. Memory OT1 and memory P14 were also transferred into naïve WT host to calculate fold expansion over OT1/P14 “take”. Popliteal LNs (pLN) were harvested at time of euthanasia (CO_2_ followed by cervical dislocation) and processed at indicated time points. **b** Representative flow cytometric plots of co-transferred memory P14 and memory OT1 fold expansion transferred at 1:1 ratio. **c** Memory OT1 (CD45.1/1) and P14 (CD45.1/2) were co-transferred into immunized mice (CD45.2/2) 1 day before VV-WR as in (**a**). **c** The fold expansion from plots shown in (**b**) was calculated as the total number of memory OT1 or memory P14 in antigen-bearing mice over the total number of memory OT1 or memory P14 in the naïve WT host (to accommodate for differences in ratio and “take”) at each respective time point. Red squares represent memory OT1s and green circles represent memory P14s transferred into the same mouse that received ova/polyI:C/αCD40 immunization 13 days prior and PBS (vehicle) 1 day after (D14). Blue squares represent memory OT1s and orange circles represent memory P14s transferred into the same mouse that received ova/polyI:C/αCD40 immunization 13 days prior and VV-WR 1 day after (D14). Each line is an individual mouse where both cell types were transferred into the same mouse. In each experiment, at least *n* = 3 mice per group were evaluated and the experiment was repeated *n* = 2 times. In each case, a different congenic marker was used for transferred cells (e.g. OT1 was CD45.1/1 and P14 was CD45.1/2 and hosts were CD45.2/2 or OT1 was CD45.1/2 and P14 was CD45.1/1 and host was CD45.2/2). Results were similar across congenic marker combinations used. In Fig. 4b, representative flow plots from one experiment are shown as an example (in the other experiment, OT1 were CD45.1/2 and P14 were CD45.1/1). Shown in Fig. 4c is the combined data from both experiments. A third replicate was not performed as our experiments were adequately powered to provide statistical significance in accordance with our IACUC policies regarding animal experiments with consistent data points. Statistical analysis was done using a paired *t*-test. Error bars are mean ± standard error of the mean. ns not significant, *p < 0.05, ***p < 0.001.

As T cells, particularly memory T cells, are able to proliferate in response to cytokine production, termed “bystander activation”^69^ in the absence of TCR ligation, we asked if the increased T cell proliferation in Fig. 3 was a result of bystander activation. To address this, we transferred either naïve OT1 or gBT T cells into congenically distinct recipient mice 1 day prior to VV-WR infection (Supplementary Fig. 6a, b). We found that while the naïve OT1 T cells (ova immunization specific) divided more at each time point after VV-WR infection compared to those that did not receive VV-WR, however the naïve gBT T cells (non-ova immunization specific) failed to divide both with and without VV-WR infection (Supplementary Fig. 6c, d). Therefore, naïve ova specific T cell division, but not HSVgB specific T cell division is significantly increased when mice were immunized with ovalbumin/polyI:C/αCD40 and then infected with VV-WR, particularly at time-points when we find increased LEC apoptosis and increased endogenous ova specific T cell responses. These findings might suggest that T cell receptor recognition of antigen, rather than cytokine derived bystander activation, mediated increased T cell proliferation and activation. However, because memory CD8 + T cells respond more readily than naïve CD8+ T cells to both lower levels of antigen and high cytokine (IL-15, IFNαβ^51,54,55^) there was a possibility that the enhanced endogenous memory CD8 + T cell activation and division were not antigen-specific but merely due to bystander activation^69^. Therefore, we asked if memory antigen specific transferred CD8 + T cells expanded as a result of antigen availability (T cell receptor engagement) or a highly inflammatory environment due to VV-WR infection (bystander activation). To do this, mice were immunized with ova/polyI:C/αCD40, memory OT1 or P14 T cells transferred 2 weeks later and 1 day later infected with VV-WR (Fig. 4a, b). We chose P14 in this experiment because the T cell receptor affinity of both OT1 and P14 T cells is high^70,71^. In line with published findings that bystander activation occurs in the presence of infection, but not necessarily due to the presentation of cognate antigen^51,72^, we found that the memory P14 T cells expanded as a result of VV-WR infection (Fig. 4c). When we compared the magnitude of expansion of transferred memory OT1 T cells to the expansion of the P14 T cells following VV-WR infection, we found a significant increase in the fold expansion of memory OT1 compared to memory P14 in mice at all time points (Fig. 4c). It appeared that the largest increase in bystander activation occurred at day 14 post-VV-WR infection based on the increased memory p14 expansion. However, at day 21 we found limited T cell expansion by transferred P14 memory cells post VV-WR and a significant increase in memory OT1 cells. These data indicate that although there is an element of bystander activation attributed to VV-WR infection, particularly at 14 days post-VV-WR, bystander activation is transient and increased proliferation subsides after 21 days. These findings suggest that archived antigen presentation following VV-WR infection leads to a predominantly antigen-specific endogenous memory CD8 + T cell response. Although there are still minor levels of activated non-antigen specific T cells, we show a significantly greater expansion of archived antigen specific memory T cells consistent with the time frame of LEC apoptosis following VV-WR infection.

### Antigen specific CD8 + T cells activated during vaccinia infection are increased in frequency and produce more cytokine following the rechallenge of previously archived antigens

**Fig. 5:**
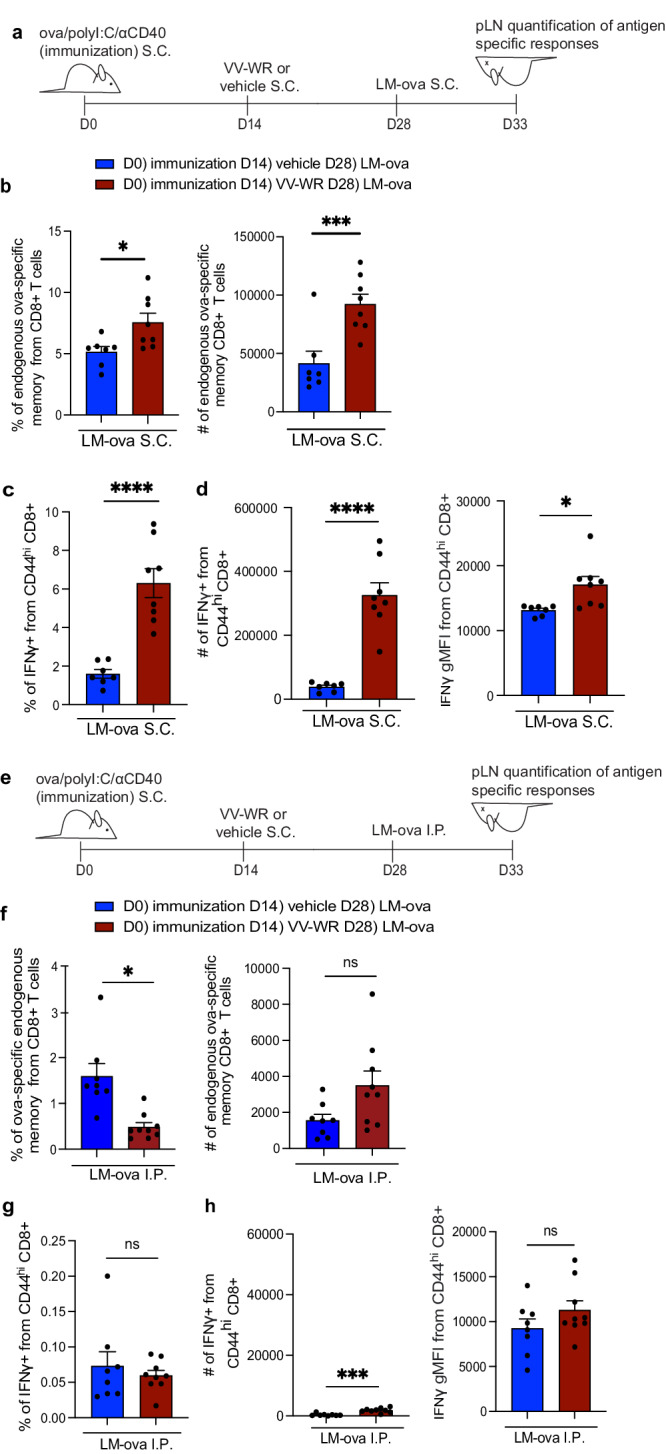
Archived antigen-specific memory CD8 + T cells derived from immunization are recalled during antigenic challenge. (**a**) Experimental schematic for (**b**–**d**). Mice were immunized with ova/polyI:C/αCD40 and infected with VV-WR 2 weeks later. Two weeks after VV-WR, mice were challenged with LM-ova subcutaneously (S.C.). Five days post-LM-ova mice were euthanized (CO_2_ followed by cervical dislocation) and popliteal lymph nodes (pLN) were harvested to assess endogenous archived-antigen (ova)-specific memory CD8 + T cells in the draining pLN. **b** Quantification of frequency and the total number of ova-specific endogenous memory CD8 + T cells in the popliteal LN as assessed by a SIINFEKL specific MHC class I K^b^ tetramer. **c** Quantification of frequency of IFNγ -produced from CD44^hi^ CD8 + T cells in the draining popliteal lymph node. **d** Quantification of the total number and geometric mean fluorescence intensity (gMFI) of IFNγ from CD44^hi^ CD8 + T cells in the draining lymph node. **e** Experimental schematic for (**f**–**h**). Mice were challenged with LM-ova intraperitoneally (I.P.) **f** Same as (**b**). except for the mice were challenged with LM-ova I.P. **g** Same as (**c**) except for the mice were challenged with LM-ova I.P. **h** Same as (**d**) except for the mice were challenged with LM-ova I.P. Statistical analysis was done using an unpaired *t*-test where the *p*-value between vaccine + vehicle + LM-ova (blue bar) and vaccine + VV-WR + LM-ova (red bar) is <0.0001. Errors bars are mean ± standard error of the mean. In (**a**–**d**) and (**e**–**h**) *n* = 3–5 mice per group were evaluated and the experiment was repeated *n* = 2 times. Shown is the combined data from both experiments. A third replicate was not performed as our experiments were adequately powered to provide statistical significance in accordance with our IACUC policies regarding animal experiments with consistent data points. ns not significant, *p < 0.05, ***p < 0.001, ****p < 0.0001.

We previously identified that archived antigens enhance protective immune responses by increasing IFNγ and IL-2 production by CD8+ T cells during antigenic rechallenge^28^. Thus, we next asked if mice with archived antigens that received an inflammatory stimulus (VV-WR) were better protected against an antigenic re-challenge. To this end, mice previously immunized with ova/polyI:C/αCD40, that did or did not receive VV-WR 2 weeks later, were challenged with a recombinant strain of *Listeria monocytogenes* that expresses ovalbumin (LM-ova) either locally (subcutaneously in the footpad) (Fig. 5a) or systemically (intraperitoneally) (Fig. 5e). Upon LM-ova challenge, we saw an increase in both the frequency and number of antigen-specific CD8 + T cells in the draining lymph node as assessed by SIINFEKL MHC class I tetramer (H2-Kb) staining (Fig. 5b, Supplementary Fig. 7). Additionally, we found that responding CD8 + T cells had a significantly higher frequency of IFNγ-producing cells (Fig. 5c, Supplementary Fig. 7). We also note that of the CD8+ T cells expressing IFNγ, more IFNγ was produced than their non-VV-WR infected counterparts (Fig. 5d, Supplementary Fig. 7**)**. This is consistent with published data demonstrating that antigen-specific tertiary memory CD8 + T cells display increased cytokine production compared to antigen-specific primary or secondary memory CD8 + T cells^73^. This was in contrast to the response seen during systemic infection (Fig. 5e) where there was no significant difference in the number of antigen-specific CD8+ T cells in the draining lymph node (Fig. 5f, Supplementary Fig. 7) nor in the frequency of cells producing IFNγ (Fig. 5g, Supplementary Fig. 7). The number of IFNγ-producing cells was higher, but strikingly low in number compared to the draining lymph node (Fig. 5d, h) while the amount of IFNγ produced was no different following LM-ova IP challenge as indicated by mean fluorescence intensity (Fig. 5h). Similarly, there was no difference in antigen-specific cell frequency or number or IFNγ production in the spleen of mice who were challenged either subcutaneously or intraperitoneally (I.P.) with LM-ova (Supplementary Fig. 8). These findings establish that antigen-specific CD8 + T cells derived from the immunization are recalled locally during a pathogenic rechallenge following an unrelated inflammatory stimulus (VV-WR) as seen by increased numbers of responding ova-specific CD8 + T cells that possess the ability to produce high levels of IFNγ (Fig. 5d, f).

### Vaccinia infection within the duration of antigen archiving induces robust and durable protective immunity

**Fig. 6:**
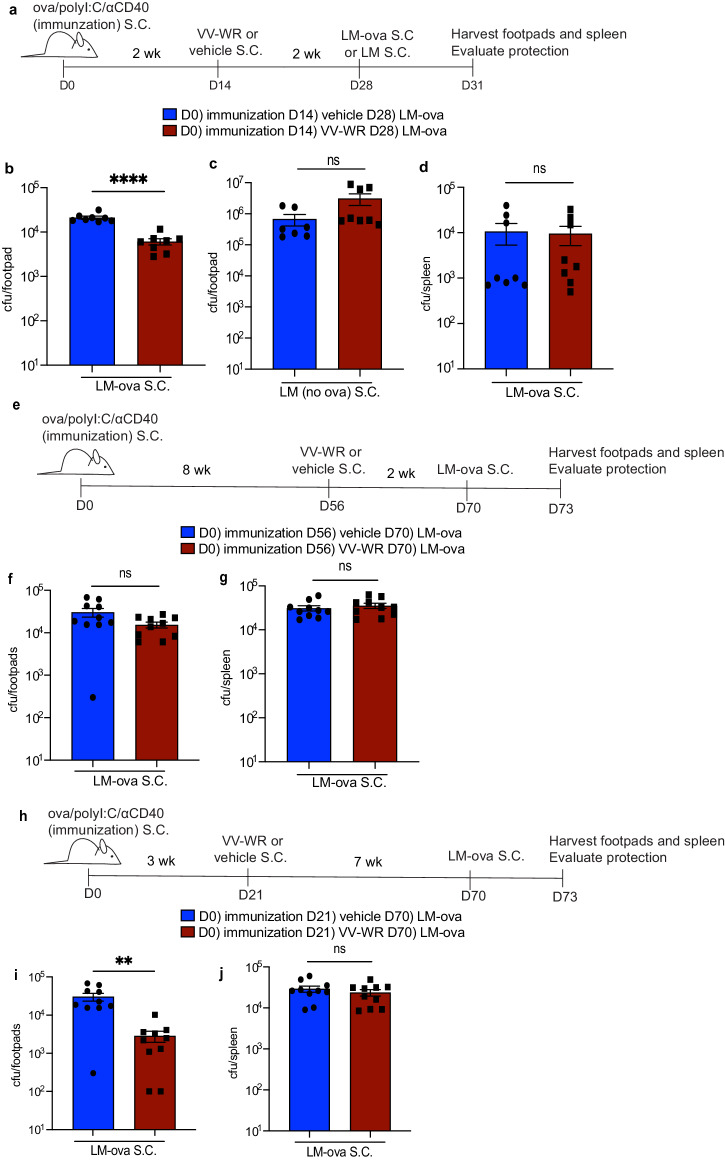
Archived antigen-specific memory CD8 + T cells derived from immunization improve protective immunity during antigenic rechallenge over the time-frame of antigen archiving. **a** Experimental schematic for (**b**–**d**). Mice were immunized with ova/polyI:C/αCD40, infected with VV-WR, and challenged with LM-ova or LM at indicated time points. Foot and ankle skin or spleen were harvested after mice were euthanized (CO_2_ followed by cervical dislocation). **b**–**d** Respective tissues were processed as described in the methods section. Homogenized tissues were plated on BHI + erythromycin (LM-ova) or streptomycin (LM) plates and colonies were counted after 3 days of growth. **e** Experimental schematic for (**f**, **g**). Mice were immunized, infected with VV-WR, and challenged with LM-ova at indicated time points. **f**, **g** Same as (**b**–**d**). **h** Experimental schematic for (**i**, **j**). Mice were immunized, infected with VV-WR, and rechallenged with LM-ova at indicated time points. **i**, **j** Same as (**b**–**d**). Statistical analysis was done using unpaired *t*-test where the *p*-value between vaccine + vehicle (blue bar) and vaccine + VV-WR (red bar) is <0.0001. Errors bars are mean ± standard error of the mean. In each experiment, at least *n* = 3–5 mice per group were evaluated and the experiment was repeated *n* = 2 times. Shown are all data points from both experiments. A third replicate was not performed as our experiments were adequately powered to provide statistical significance in accordance with our IACUC policies regarding animal experiments with consistent data points. ns not significant, **p < 0.01, ****p < 0.0001.

We next asked if the increase in the number of CD8 + T cells with enhanced effector function limited bacterial burden at the site of bacterial infection after VV-WR infection (Fig. 6a). Indeed, ovalbumin immunized mice infected with VV-WR and then rechallenged with LM-ova demonstrated a small, but significant and repeatable reduction in colony-forming units (CFU) of LM-ova in the skin of the footpads compared to mice that did not receive VV-WR (Fig. 6b). This protective phenotype was dependent on the originally archived antigen (ova) as we did not detect a significant difference in CFU from mice infected with LM that did not express ova regardless of whether they were infected with VV-WR or not 2 weeks prior (Fig. 6c). In parallel with the observed T cell phenotypic and functional assays assessed after systemic LM-ova infection (Fig. 5e-h), we found no difference in protection in the spleen of mice infected with LM-ova either subcutaneously (Fig. 6d) or intraperitoneally (Supplementary Fig. 8g). These data suggest that memory ova-specific CD8 + T cells are primed locally during VV-WR infection and that the memory ova-specific CD8 + T cells increase protection against a homologous re-challenge (LM-ova), but not a heterologous re-challenge (LM-no ova).

Thus far we have shown that we can induce LEC apoptosis in order to facilitate activation of antigen-specific T cells to accumulate with enhanced effector cytokine responses during rechallenge to ova-expressing pathogens. We next evaluated the longevity by which these downstream memory T cell responses can occur in order to improve local protection upon encounter of a cutaneous pathogen during antigenic re-challenge. We assessed how VV-WR infection influenced downstream effector CD8 + T cell responses at a time point after archived antigen is no longer detectable. To evaluate when archived antigen was no longer available, mice were immunized with ova/polyI:C/αCD40, and 3 or 8 weeks later VPD labeled OT1 T cells were transferred into immunized mice. We found that while at 3 weeks post-subunit immunization there was robust OT1 division, at 8 weeks there was no longer OT1 division (Supplementary Fig. 9). This demonstrates that T cells could only respond to the archived antigen remaining in the lymph node for <8 weeks following ova/polyI:C/αCD40. Based on the time frame during which antigen remains archived within LECs, we infected mice with VV-WR at 8 weeks post-immunization and rechallenged the mice with LM-ova 2 weeks post-VV-WR (Fig. 6e). We saw no significant difference in bacterial burden whether or not mice were infected with VV-WR prior to the LM-ova rechallenge (Fig. 6f). There was also no significant difference in CFUs in the spleen between mice that were infected with VV-WR and non-infected mice (Fig. 6g). Thus, when local archived antigens are not available to stimulate memory CD8 + T cells during an additional inflammatory event, the protective capacity against a pathogen expressing the previously archived antigen is no longer present.

However, because we saw bystander activation peaking two weeks post VV-WR it was possible that the increased protection against LM-ova 2 weeks post VV-WR (Fig. 6b) was a result of bystander activation. Furthermore, it was possible based on DC turnover in the skin^74^ that there were a small number of DCs remaining from the initial immunization. To test this, we infected mice with VV-WR at 3 weeks post-immunization, when all DCs from the initial immunization should be gone^74^. 7 weeks later (Fig. 6h), and beyond the time frame of VV-WR-induced regulation of cytokines associated with bystander activation^51,54,55^, we evaluated protection against LM-ova in the skin and distantly in the spleen. Importantly, at 7 weeks post-VV-WR infection, the virus infection has fully resolved with the resulting cytokine profile also returning to homeostatic levels^75,76^ (Supplementary Fig. 2e). We observed a significant reduction in CFU in the footpads of mice infected with VV-WR and thus better local protection compared to mice that were not infected with VV-WR (Fig. 6i). This suggests that following VV-WR infection, the memory T cells we identified in Fig. 5 are more protective against antigenic challenge at the tissue site as a result of the recognition of their cognate antigen in the draining LN. However, we further establish that this protective phenotype, mediated by memory T cells is specific to local re-challenge as there was no increase in protection in the spleen (Fig. 6j). These findings demonstrate that archived-antigen-specific (ova) T cells can be stimulated by archived ova during a secondary inflammatory insult and that these stimulated antigen-specific T cells can maintain protective responses locally during pathogenic rechallenge in a durable manner.

## Discussion

In this study, we established a model by which we can boost cell-mediated immunity through the presentation of previously archived antigens stored in LECs. We demonstrate an increased benefit in protective immunity via the stimulation of ovalbumin immunization-specific CD8 + T cells during an antigenically unrelated infection or stimulus. We propose that the memory CD8 + T cells are boosted by the antigen archived within the LECs, during the inflammatory event, as a result of LEC apoptosis and DC activation. LEC apoptosis and activation of DCs during the inflammatory stimulus and lymph node contraction stimulate CD8 + T cells as a result of cDC1 cross-presentation (Fig. 2). This is evidenced by the increased amounts of IFNγ produced by the expanded memory CD8 + T cells following VV-WR infection as well as the increased protection seen with a lower bacterial burden during a pathogenic re-challenge with the archived antigen from the immunization. While we also show that non-antigen-specific memory T cells (P14 or gBT) expand to a degree in response to cytokine stimulation (i.e., bystander activation^51,55^), we go on to show that archived antigens are a more substantial modulator of CD8 + T cell memory activation (Fig. 4). Indeed, the difference in protection that we find is only at the tissue site where the immunization was administered. These findings suggest that the memory CD8 + T cells traffic back to the site of infection to exert cytotoxic functions locally and to protect against the insult at the site of initial infection. Furthermore, in our findings, we also identify a specific time frame by which VV-WR must be administered in order for the protective benefits of memory CD8 + T cells to occur. Beyond the time frame of antigen archiving, we do not detect any appreciable differences in bacterial load, even at the local site of immunization (Fig. 6).

In assessing the contribution of antigen archiving to multiple sequential infections, we considered an unrelated viral infection as a potential method to increase LEC apoptosis of antigen bearing LECs^29^ in addition to activating DC migration. LECs have been demonstrated to expand and contract following lymph node expansion and contraction^58,77,78^. This is an important feature for DC and neutrophil recruitment to the lymph node during infection or immunization where LECs also express the chemokine ligand CCL21^79,80^. Consistent with these findings we indeed show that LECs that have gone through the same vaccination and infection timeline do undergo increased apoptosis at the 14 and 21 day time points, and that this is independent of previous immunization (Fig. 2 and Supplementary Fig. 2). This timing is consistent with lymph node expansion and contraction as the immune response is activated and resolved. Whether the LECs undergo apoptosis due to a return to homeostasis or as a result of viral infection is still unknown. Regarding vaccinia infection, while the cell entry receptor for vaccinia virus is not well defined, there is evidence that the scavenger receptor MARCO contributes to viral entry into keratinocytes. Since some LEC subsets express MARCO it is possible that one mechanism of apoptosis is through vaccinia infection of the LECs. However, we have been unable to detect virus within primary murine LEC cultures. Furthermore, the specific process and mechanism by which antigen exchange occurs between LECs and DCs remains unclear. A possibility is that apoptotic LECs release extracellular vesicles^81^, and these vesicles undergo uptake by migratory cDC1s that encounter these apoptotic bodies within the subcapsular sinus of the LN (Fig. 2). Additional mechanisms may include DC trogocytosis or cytoplasm exchange^82^ of archived antigens from LECs^83^, in order to facilitate DC acquisition of archived antigens. These processes may happen to a lesser degree in the absence of infection or inflammation to maintain a long-lasting memory CD8 + T cell pool that can rapidly respond to pathogenic infection occurring within distal sites of the lymph node^84^ and exert cytotoxic functions as we have shown^28^.

Data shown here suggests that the addition of an inflammatory stimulus to the process of archived antigen exchange between LECs and DCs could be affecting this mechanism in a number of ways. One mechanism could be that the inflammatory stimulus may increase antigen release from LECs to further enhance acquisition and presentation by DCs. A second possibility is that the inflammatory stimulus could also increase the frequency of LEC-DC interactions as certain inflammatory stimuli increase the amounts of migratory DCs arriving in the draining lymph node from the site of the infection or inflammatory stimulus. A third possibility could be that the inflammatory stimuli further activate resting LN-resident DCs to provide the required cytokines and co-stimulatory molecules. This would provide the necessary signal for the responding CD8 + T cells to establish a secondary memory function i.e. increased IFNγ production upon rechallenge^56,57^.

The divergent pathways that allow for memory CD8 + T cells to have a superior ability to control pathogens during secondary infection through increased proliferation and elaboration of effector cytokines, especially during the time frame of antigen archiving, is still unknown. However, based on these studies, it would be pertinent to evaluate which kind of adjuvant would be most beneficial in promoting T-cell mediated immunity in order to initiate the most robust and durable CD8 + T cell memory response to protect against severe disease and pathogens. A prime example of this is the observed T cell memory responses evaluated following the SARS-CoV-2 mRNA lipid nanoparticle (LNP) vaccination where protection by T cells against severe disease happens in the face of waning antibody titers, which has been critical for patient survival^85^. As mentioned above, the process of antigen archiving by LECs and possibly by other lymph node stromal cells (such as follicular dendritic cells and or other fibroblastic reticular cell subsets) appears to be most beneficial to the host during the memory phase of the immune response. As such, it is important to further characterize which types of currently available vaccines are able to induce antigen archiving and which specific properties of LECs allow them to archive antigens in a non-degraded state. To begin to fully understand which vaccine types are capable of eliciting antigen archiving, we found that all TLR agonist-adjuvanted vaccines we have tested are capable (Fig. 1 and^28,30^), but whether mRNA-based vaccines contained within LNPs, viral vector vaccines, virus-like particles or others result in antigen archiving by lymph node stromal cells is currently unknown. However, it has been reported that virus-like particles can be detected in the draining lymph node for up to 6 days after intradermal injection in a mouse model using an infrared dye^86^. Further, mRNA from Sars-CoV-2 mRNA vaccination was demonstrated to persist in the axillary draining lymph node for up to 26 days in some patients^87^. The Sars-CoV-2 spike protein, derived from the mRNA vaccination, was detected in the lymph node of vaccinated people for up to 60 days. These data may suggest that lymph node stromal cells (eg follicular dendritic cells, lymphatic endothelial cells) may be able to retain protein antigens within the lymph node following mRNA vaccination^88^. The exact cell type holding the mRNA or the protein antigen derived from the mRNA was not determined. Whether lymph node stromal cells (eg follicular dendritic cells, lymphatic endothelial cells or fibroblastic reticular cells) are able to archive antigens to benefit protective vaccine mediated immunity in humans has yet to be demonstrated. Future study into lymph node stromal cell antigen archiving in humans is needed to fully understand the importance of antigen archiving.

Many current vaccines utilize aluminum salt (alum) as an immune adjuvant, which has been successful at initiating robust antibody-dependent responses to the antigen administered with the help of CD4 + T cells, however, cell-mediated immunity through robust CD8 + T cell responses are minimal with these current vaccine strategies^89^. It is unlikely that the antigen administered with alum is archived like subunit vaccines, but rather forms an antigen depot, perhaps unimportant for the immune response^90^, at the injection site rather than a bolus of antigen that can be received by the LECs within the draining LN. Furthermore, as we have also published that a concomitant T cell response is required for antigen archiving, it seems unlikely that alum provides the same protective benefit due to the minimal T cell response^28^, however, this has yet to be tested. Future studies should be aimed at investigating how LECs and other lymph node stromal cells are capable of archiving non-degraded antigens and maintaining them for extended periods of time and whether these findings can be translated into humans. Our single-cell sequencing analysis revealed that the genes *Cavin1* and *Cavin2* were upregulated in antigen-positive LECs^30^, but not in hematopoietic populations. Previous literature has established that caveolin-mediated endocytosis depends on Caveolin1 (CAV1) and Caveolin2 (CAV2) at the membrane, which interact with Cavin1 (CVN1) and Cavin2 (CVN2) to stabilize caveolae^91^. These findings support a possible model where LECs retain antigen in non-degradative endosomes over long periods of time^92^, unlike DCs, because caveolin-mediated endocytosis differs from pinocytosis, macropinocytosis, receptor-mediated endocytosis, and phagocytosis, in that caveosomes are specially equipped to retain endocytosed proteins. Caveosomes maintain a neutral pH, with cargo able to remain in caveosomes until either transcytosis/recycling or lysosomal degradation via RAB5-dependent fusion with the early endosome^93^. Indeed, we found that blocking caveolin-mediated endocytosis with nystatin led to a significant decrease in antigen acquisition by LECs in vivo^30^. Our findings using single-cell mRNA sequencing analysis revealed caveolin-mediated endocytosis proteins to be upregulated at both early and late time points during the timeframe in which LECs are antigen-positive^30^. We are currently investigating whether we can skew LECs toward caveolin-mediated endocytosis as a means to prolong antigen archiving and thus achieve a more durable and lasting memory T cell response that we observe within this study upon reinfection. These studies inform vaccine design, specifically geared at improving memory CD8 + T cell response to vaccination.

Antigen archiving or persistence by lymph node stromal cells has thus far only been shown to benefit protective immunity^35^. However, lymph node stromal cells are also major contributors to the maintenance of peripheral tolerance via expression of peripheral tissue antigens in both mice and humans^48,49,94–98^. In mice LECs can induce T cell tolerance through PD-L1 expression when foreign antigens are administered in the absence of an immune adjuvant^50^. It is clear that self antigens or foreign antigens presented by MHC class I on lymph node stromal cells can result in immune tolerance^49^. However, data presented here (Fig. 2) and^28,29^ suggests that to provide memory T cells with increased function, archived antigens must be presented by dendritic cells. However, whether lymph node stromal cells may also use this process of antigen acquisition and retention to mute allergic responses in the absence of inflammation is still something that should be considered.

Collectively, here and in our prior work we have demonstrated that antigen archiving provides a unique purpose in enhancing memory CD8 + T cell function, particularly when an unrelated inflammatory stimulus is involved in order to further enhance and drive memory T cell response upon reinfection at a local site, such as the skin. While we do not claim that antigen archiving is required for memory formation or maintenance, as was previously demonstrated^99^. We provide an important purpose for antigen archiving in enhancing T cell-mediated immunity and exemplify how non-canonical immune cells, like LECs, contribute to vaccine-elicited immunity and encourage protection against antigenically related pathogens. We speculate that these findings may have application to vaccines, particularly those that use toll like receptor mediated adjuvants. This may be an additional factor to consider when determining optimal immunization platforms in humans. Together, the findings outlined in this manuscript are important to consider when evaluating immune memory, particularly CD8 + T cell memory following vaccination or viral infection. Despite the difficulty in evaluating stromal cells of the lymph nodes of people, these animal studies demonstrate a unique function for lymph node stromal cells to immunity, and potential avenues that could be considered to employ lymph node stromal cells or LEC functions to improve vaccine-mediated immunity. Future studies should be focused on whether human LECs or other human lymph node stromal cells provide the same protective benefit outlined in these mouse models.

## Methods

### Mice

All animal procedures were approved by and in accordance with the Institutional Animal Care and Use Committee at the University of Colorado Anschutz Medical Campus under protocol number 67. 5–8 week-old male or female mice were purchased from Charles River or Jackson Labs and used at ages between 6 and 10 weeks and were bred and housed in the University of Colorado Anschutz Medical Campus Animal Facility. All animals were euthanized with carbon dioxide (CO_2_) followed by cervical dislocation prior to necropsy. Wild type, *Karma*, OT1, P14, and gBT mice were all bred on a C57BL/6 background. OT1 mice are a TCR transgenic strain specific to the SIINFEKL peptide of ova (OVA257-264) in the context of H-2K^b^. P14 mice are a TCR transgenic strain specific to the gp33 peptide. gBT-1 (gBT) mice are a TCR transgenic strain specific to the SSIEFARL peptide of herpes simplex virus glycoprotein B (HSV-1 gB 498-505) in the context of H-2K^b^. No differences in sex or age were found in experiments.

### Vaccines and pathogen challenge

Mice were immunized subcutaneously in each footpad with the indicated protein antigen (amount administered in parenthesis), 5 μg polyI:C, and 5 μg αCD40. Ova (10 μg) was purchased from Sigma-Aldrich (Cat No. A5503) and Chikungunya virus envelope 2 protein (8 μg) (CHIKV-E2, strain SL-CK1) was purchased from Sino Biological (Cat. No. 40440-V08B). HSVgB-BSA (10 μg) was made by combining 10 mg of maleimide-activated bovine serum albumin (BSA) (Thermo Fisher Cat. No. 77115) with 15 mg of SSIEFARL gBT peptide for 2 h at room temperature. The conjugated HSVgB-BSA was enriched and concentrated using a 30 kDa size exclusion column. SARS-CoV-2-RBD protein was generated by the University of Colorado Cell Technologies Shared Resource Core. SARS-CoV2-RBD (8 μg) (GenBank: MT380724.1) was made by transfecting HEK293 T cells with a His-tagged vector and the protein was purified over ATKA nickel column. For immunization with fluorescent antigens, ova, HSVgB-BSA, SARS-CoV-2-RBD, and CHIKV-E2 were conjugated to AlexaFluor-488 via NHS Ester kit (Thermo Fisher Cat. No. A20000). Ova-psDNA (10 μg) was created as previously described^30^ with the addition of a fluorescein molecule conjugated to the psDNA (ova-psDNA-6FAM) for visualization using flow cytometry. Endotoxin levels were determined using the amebocyte lysate method using a Pierce™ Chromogenic Endotoxin Quant Kit (Thermo Scientific, Cat. No. A39553) to be <1 endotoxin unit (EU) per milligram of protein. When necessary endotoxin removal was performed using the protocol from Aida & Pabst^100^. Briefly, 40 mg/mL of protein in PBS was incubated with 1% Triton X-114 (Sigma Aldrich, Cat. No. X114) on ice for 5 min, then 37 °C for 5 min. The mixture was spun at 2095 x g with no brake for 5 min at room temperature, and the top layer was collected. This process was repeated for a total of three times. To remove excess Triton from the endotoxin-depleted protein, the depleted protein was incubated with hydrophobic Bio-Beads SM-2 Adsorbents (Bio-Rad, Cat. No. 1523920) overnight at 4 °C. For the viral challenge, mice were infected with 10^4^ plaque-forming units (pfu) per footpad of Vaccinia Virus Western Reserve strain. For subcutaneous re-challenge with *Listeria monocytogenes* (LM) or LM expressing ova (LM-ova), the bacteria were grown in Brain Heart Infusion media from a frozen stock overnight with streptomycin (LM) or erythromycin (LM-ova) and sub-cultured for 1–4 h until the bacterial culture reached an optical density (OD) at 600 nm wavelength of 0.3–0.5. Calculating 1E9 per 1.0 OD, mice were injected with 5e5 per footpad in 50 μl or 2E5/mouse in 200 μl.

### Tetramer and intracellular cytokine staining

Draining LNs and spleens were harvested and processed by frosted glass slide maceration. Red blood cells from the spleens were lysed using Ammonium-Chloride-Potassium (ACK) lysis buffer. The cells were filtered, washed, and suspended in complete RPMI with 2.5% fetal bovine serum (FBS). Cells were stained with anti-mouse CD8 antibody (clone: 53-6.7, APC-Cy7, 1:300 dilution, Biolegend Cat. No. 100714) and both SIINFEKL tetramer-PE and SIINFEKL tetramer-APC (NIH tetramer core facility) for 1 h at 37 °C. Cells were then stained for additional surface markers (CD3 BV510 1:200 (Biolegend Cat. No. 100233), CD4 PerCP 1:200 (Biolegend Cat. No. 100537), CD69 PE-cy7 1:200 (Biolegend Cat. No 104512), CD44 PacBlue 1:400 (Biolegend Cat. No 103019) or CD44 RF710 1:200 (Tonbo Cat. No. SKU 80-0441-U100) or CD44 PerCP Cy5.5 1:300 (Biolegend Cat. No. 103032), B220 BV421 1:200 (Biolegend Cat. No. 103251) – see Table 1 for clone numbers) for 30 min at 37 °C. After washing, samples were run on BD Canto II flow cytometer or Beckman Coulter Cytoflex LX flow cytometer. For intracellular cytokine staining, single-cell suspensions were ex vivo stimulated in brefeldin A (1 μg/ml) with or without (2 μg/ml) SIINFEKL peptide for 4–6 h at 37 °C. After stimulation, cells were stained with anti-CD8 APC-Cy7 (1:300, Biolegend Cat. No. 100714), B220 BV421 1:200 (Biolegend Cat. No. 103251), CD3 BV510 1:200 (Biolegend Cat. No. 100233), and CD44 RF710 1:200 (Tonbo Cat. No. SKU 80-0441-U100) or CD44 PerCP Cy5.5 1:300 (Biolegend Cat. No. 103032) antibodies (see Table 1 for clone numbers). Cells were then fixed with 1% paraformaldehyde and 3% sucrose for 10 min in the dark at room temperature. Cells were washed twice with FACS buffer (0.1% bovine serum albumin (BSA), 1x Hank’s buffered saline solution, 2 mM ethylene diamine tetra acetic acid (EDTA) and 0.02% sodium azide) and then permeabilized with 1x perm wash (BD Cat. No. 554723). The cells were then stained for IFNγ (clone: XMG1.2) in 1x perm wash. The following day, the cells were washed in perm buffer 2 times and resuspended in FACS buffer before acquiring on either a Canto II (BD biosciences) or Cytoflex LX (Beckman Coulter). All flow cytometry data were analyzed with FlowJo software and statistical analysis and graphing was done using Graphpad Prism software. See the list of antibodies used in the table for reference.

**Table 1:** Antibodies/reagents

Reagent Type	Designation	Source or reference	Clone	Additional information
Chemical compound	Violet proliferation dye	BD Biosciences	-	-
Chemical compound	CFSE	BD Biosciences	-	-
Chemical compound	PolyI:C	Invivogen	-	subcutaneous injections, 5 μg/mouse; intraperitoneal injections, 50 μg /mouse
Antibody	Anti-mouse CD40 (Rat monoclonal)	BioXcell	FGK4.5	subcutaneous injections, 5 μg/mouse; intraperitoneal injections, 50 μg/mouse
Antibody	Anti-mouse CD8 (Rat monoclonal)	Biolegend	53–6.7	Dilution – 1:200-1:300
Antibody	Anti-mouse/human B220/CD45R (Rat monoclonal)	Biolegend	RA3-6B2	Dilution - 1:200
Antibody	Anti-mouse CD3 (Rat monoclonal)	Biolegend	17A2	Dilution - 1:200
Antibody	Anti-mouse CD4 (Rat monoclonal)	Biolegend	RM4-5	Dilution - 1:200-1:300
Antibody	Anti-mouse CD69 (Armenian Hamster monoclonal)	Biolegend	H1.2F3	1:200
Antibody	Anti-mouse CD44 (Rat monoclonal)	Tonbo	IM7	Dilution - 1:200
Antibody	Anti-mouse CD44 (Rat monoclonal)	Biolegend	IM7	Dilution - 1:200-1:400
Antibody	Anti-mouse CD19 (Rat monoclonal)	Biolegend	6D5	Dilution 1:200
Antibody	Anti-mouse IFNγ (Rat monoclonal)	Biolegend	XMG1.2	Dilution - 1:200
Antibody	Anti-mouse Vβ5 (Mouse monoclonal)	Biolegend	MR9-4	Dilution - 1:200
Antibody	Anti-mouse Vβ8 (Rat monoclonal)	Biolegend	KJ16-133.18	Dilution - 1:200
Antibody	Anti-mouse CD45.1 (Mouse monoclonal)	Biolegend	A-20	Dilution - 1:300
Antibody	Anti-mouse CD45.2 (Mouse monoclonal)	Biolegend	104	Dilution - 1:200
Antibody	Anti-mouse CD45 (Rat monoclonal)	Biolegend	30-F11	Dilution - 1:300
Antibody	Anti-mouse CD31 (Rat monoclonal)	Biolegend	390	Dilution - 1:200
Antibody	Anti-mouse PD-L1 (Rat monoclonal)	Biolegend	10 F.9G2	Dilution – 1:200
Antibody	Anti-mouse podoplanin/gp38 (Syrian hamster monoclonal)	Biolegend	8.1.1	Dilution - 1:200
Antibody	Anti-mouse CD11c (Armenian Hamster monoclonal)	Biolegend	N418	Dilution – 1:400
Antibody	Anti-mouse MHC Class II (I-A/I-E) (Rat monoclonal)	Biolegend	M5/114.15.2	Dilution – 1:1000
Antibody	Anti-mouse XCR1 (Mouse monoclonal)	Biolegend	ZET	Dilution – 1:200
Mouse strain, background (*Mus musculus*)	WT – C57BL/6	Charles River Labs or Jackson Labs	–	–
Mouse strain, background (*Mus musculus*)	OT1 - C57BL/6-Tg(TcraTcrb) 1100Mjb/J	Jackson Labs	–	–
Mouse strain, background (*Mus musculus*)	P14	Kind gift from Raul Torres, Univeristy of Colorado Anschutz	–	–
Mouse strain, background (*Mus musculus*)	gBT-1 (gBT)	Kind gift from Bill Heath, University of Melbourne	–	–
Mouse strain, background (*Mus musculus*)	*Karma*	Kind gift from Marc Dalod, Centre d’Immunologie de Marseille-Luminy (CIML)	–	–

### Stromal cell harvesting and staining

Draining LNs were harvested into Click’s EHAA media (FUJIfilm) and minced with 22-gauge needles. Tissues were digested in 0.25 mg of liberase dispase low (DL) (Sigma-Aldrich Cat. No. 5466202001) and 17 μg/ml DNAse (Worthington Biochemical Cat. No. LS002145) for 1 h at 37 °C with pipetting every 15 min to physically agitate the digested tissues. Following digestion, cells were filtered through a 100-micron screen and washed with 5 mM EDTA and 2.5% FBS in EHAA media to stop the digestion. Cells were washed once with PBS before staining in live/dead GhostRed stain for 30 min at 4 °C. Cells were then washed with FACS buffer and stained with anti-mouse CD45 APC-Cy7 1:300 (Biolegend Cat. No. 103116), CD31 BV785 1:200 (Biolegend Cat. No. 102435), and podoplanin APC 1:200 (Biolegend Cat. No. 127409) and PD-L1 PeCy7 1:200 antibodies (Biolegend Cat. No. 124314) in 10% 24G2 (Fc Block) for 30 min at 4 °C. Cells were washed twice with FACS buffer and run on BD Canto II flow cytometer or Beckman Coulter Cytoflex LX flow cytometer.

### Protection assay

Footpads and spleens were harvested in 0.2% NP-40 in PBS. The spleen was mascerated mechanically by grinding between two frosted glass slides. The skin of each footpad was removed from the bones and homogenized with a tissue homogenizer. Homogenized tissues were diluted 1:10, 1:1000, 1:10,000 with PBS. All dilutions were either plated onto Bacto-Brain Heart Infusion (BHI) plates with 5 μg/mL erythromycin for LM-ova selection or plated on BHI with 50 ug/mL streptomycin for LM selection. Plates were incubated at 37 °C for 1–3 days and colonies were counted.

### OT1, gBT, and P14 isolation and transfer

OT1, gBT, and P14 CD8 + T cells were isolated using the Mojosort CD8 T cell isolation kit (Biolegend Cat. No. 480008). After CD8 negative selection, the cells were labeled with VPD or CFSE to assess proliferation. For generating memory OT1 or memory P14, naïve T cells were isolated as described above, 1e5 cells were intravenously transferred into WT mice of a different congenic background and the following day the immunized mice were intravenously injected with the following to expand each respective transgenic T cells: 100 μg ova, 50 μg polyI:C, and 50 μg anti-CD40 for memory OT1; 100 μg gp33 peptide, 50 μg polyI:C, and 50 μg anti-CD40 for memory P14. After 2–4 weeks, generated memory CD8 + T cells were isolated from the mice and isolated by negative selection using Mojosort CD8 T cell isolation. Antigen-specific CD8+ cell frequency and number were quantified with respective tetramers (NIH core tetramer facility) by flow cytometry and ~8E5-1E6 cells were transferred at a 1:1 ratio into immunized mice as described in Fig. 4a.

### DT depletion

Diphtheria toxin from *Corynebacterium diphtheriae* (DT) (Sigma Aldrich Cat. No. D0564-1MG) was resuspend in sterile distilled water to a stock concentration of 2 mg/mL. Mice were injected with 32 ng/g of DT intraperitoneally 1x at day 11. Mice were then dosed at 16 ng/g starting at day 13 every day for 7 doses and then every other day starting at day 22 for 4 doses until euthanasia with carbon dioxide followed by cervical dislocation (1 day following the last DT dose) at day 28 for a total of 12 DT injections (refer to Fig. 2 for outline).

### Dendritic cells (DC) harvesting and staining

Draining lymph nodes from DT-depleted *Karma* mice were harvested into Click’s EHAA media (FUJIfilm) and minced with 22-gauge needles. Minced tissues were digested with 100 μg/ml collagenase D (Millipore Sigma Cat No. 11088882001) and 17 μg/ml DNAse (Worthington Biochemical Cat. No. LS002145) for 30 min at 37 °C. Following digestion, cells were filtered through a 100-micron screen and washed with 5 mM EDTA and 2.5% FBS in EHAA media to stop the digestion. Cells were washed with FACS buffer and stained with B220 FITC 1:200 (Biolegend Cat. No. 103206), CD11c APC Cy7 1:400 (Biolegend Cat. No. 117324), MHC Class II (I-A/I-E) BV421 1:1000 (Biolegend Cat. No. 107631), and XCR1 BV785 1:200 (Biolegend Cat. No. 148225) or XCR1 BV650 1:200 (Biolegend Cat. No. 148220) antibodies in 10% 24G2 (Fc Block) for 30 min at 4 °C. Cells were washed twice with FACS buffer and run on BD Canto II flow cytometer or Beckman Coulter Cytoflex LX flow cytometer.

### Antigen-Specific ELISA

High protein-binding microtiter plates (Costar) were coated with ovalbumin at 2 μg/ml in phosphate-buffered saline (PBS) overnight at 4 °C. Plates were washed with PBS/0.05% Tween and blocked with PBS containing 20% fetal bovine serum (FBS) for 1 h at 37 °C. Sera were then serially diluted 1:50 and serially diluted 3-fold. Diluted sera were transferred to blocked ELISA plate and incubated for 1.5 h at 37 °C. Horseradish peroxidase (HRP)-conjugated goat anti-mouse IgG (1, 2b, 2c, 3) antibody diluted 1:1000 (Jackson ImmunoResearch) was added to washed plates and were subsequently developed with Super Aquablue ELISA substrate (Invitrogen). Absorbance was measured at 405 nm on a microplate spectrophotometer (BioRad). End point titers were extrapolated from sigmoidal 4PL (where X is log concentration) standard curve for each sample using Graphpad Prism (v10). The threshold to calculate the end point titers was the mean plus 2 standard deviations of naïve mouse sera on the ELISA plate as a given sample.

### Caspase 3/7+ staining

Tissues were harvested and processed as “Stromal Cells Harvesting and Staining” in the Methods section. Cells were then washed with FACS buffer and stained with CD45 BV510 1:300 (Biolegend Cat. No. 103138), CD31 PerCP Cy5.5 1:200 (Biolegend Cat. No. 102420), and podoplanin APC 1:200 (Biolegend Cat. No. 127409) antibodies in 10% 24G2 (Fc Block) for 30 min at 4 °C. Cells were washed with FACS buffer and stained with CellEvent Caspase-3/7 Green Flow Cytometry Assay kit (Thermo Fisher Cat. No. C10427) for 25 min at 37 °C. Cells were run onto the cytometer without washing. In some experiments, in addition to caspase 3/7, and prior to staining with surface antibodies, cells were washed once with PBS before staining in live/dead GhostRed stain (Tonbo Bioscences Cat. No. 50-105-2988) for 30 min at 4 °C.

### Statistical analysis

Comparison of the means between samples using an unpaired Student’s *t*-test, paired Student’s *t*-test and two-way ANOVA in Graphpad Prism 9 were performed. *p*-values are denoted in the figure legends and in the figure images, where one asterisk represents a *p*-value of <0.05 and two asterisks a *p*-value of <0.01, three asterisks a *p*-value of <0.001 and four asterisks a *p*-value of <0.0001. All *t*-tests were two-tailed. All analysis assumed both populations were normally distributed and parametric tests were used. A confidence interval of 95% was used. Each in vivo analysis was performed with 3–5 mice per group as determined by a power calculation using the assumption (based on prior data) that there will be at least a two fold change with a standard deviation of <0.5. To calculate numbers we performed a power calculation with an alpha of 0.5 and a 1-beta of 0.80 to determined at least 3 mice per group should be evaluated. Exact replicates and numbers are provided in the figure legends. Each analysis was done with at least three mice per treatment group and each experiment was done at least twice with the same results. Error bars are mean ± the standard error of the mean.

#### Viral Plaque Assay

For the viral plaque assay in Supplementary Fig. 2E, 1-1.5e6 vero cells per well were seeded in 24-well plate in 0.5 mL of complete MEM with 5% FBS overnight. Popliteal lymph nodes were harvested and homogenized with the tissues grinder. Mix homogenized tissues with 0.25% trypsin at 1:1 ratio and incubate for 37 °C for 1 h. Dilute the mixture at 1:1000 with PBS. Add 50 uL of neat or 1:1000 homogenized tissues + trypsin mixture to each well in triplicate and incubated for 2 days at 37 °C. Media was removed and 0.5 mL of 10% buffered formalin was added prior to 5 min incubation at room temperature. Formalin was aspirated 0.5 mL of 0.1% crystal violet was added (diluted in 20% ethanol). Crystal violet was aspirated and the number of plaques counted after the wells dried.

#### RBD intracellular cytokine

Experiments were performed as with ovalbumin except RBD protein was injected at 8 μg/footpad and isolated LN cells were stimulated for 6 h ex vivo with the VV *VVLSFELL peptide*.

#### Naive T cell transfer and division

OT1 and T cells were isolated using the Mojosort CD8 T cell isolation kit (Biolegend Cat. No. 480008). After CD8 negative selection, the cells were labeled with VPD or CFSE to assess proliferation. 5e5-1e6 isolated cells were transferred into immunized mice 3 days before harvest. For OT1 or gBT divisions were (percent divided) was calculated as previously described^101^ using the equation fraction diluted =∑i1Ni2i/∑i0Ni2i = ∑1iNi2i/∑0iNi2i, where *i* is the generation number (0 is the undivided population), and *N*_*i*_ is the number of events in generation *i*.

## Availability of data and material

All data and material within this article will be available upon reasonable request to the corresponding author.

### Reporting summary

Further information on research design is available in the Nature Research Reporting Summary linked to this article.

### Supplementary information

Supplemental materials

REPORTING SUMMARY

## Data Availability

All data within this article will be available upon reasonable request to the corresponding author.

## Material availability

All material within this article will be available upon reasonable request to the corresponding author.
