# Supplementary material for: Immunization-induced antigen archiving enhances local memory CD8+ T cell responses following an unrelated viral infection
Source: NPJ Vaccines. 2024 Mar 21;9:66. doi: 10.1038/s41541-024-00856-6 (PMC10957963; doi:10.1038/s41541-024-00856-6)
Supplement: Supplementary file 1 — Supplemental materials [file 41541_2024_856_MOESM1_ESM.pdf]

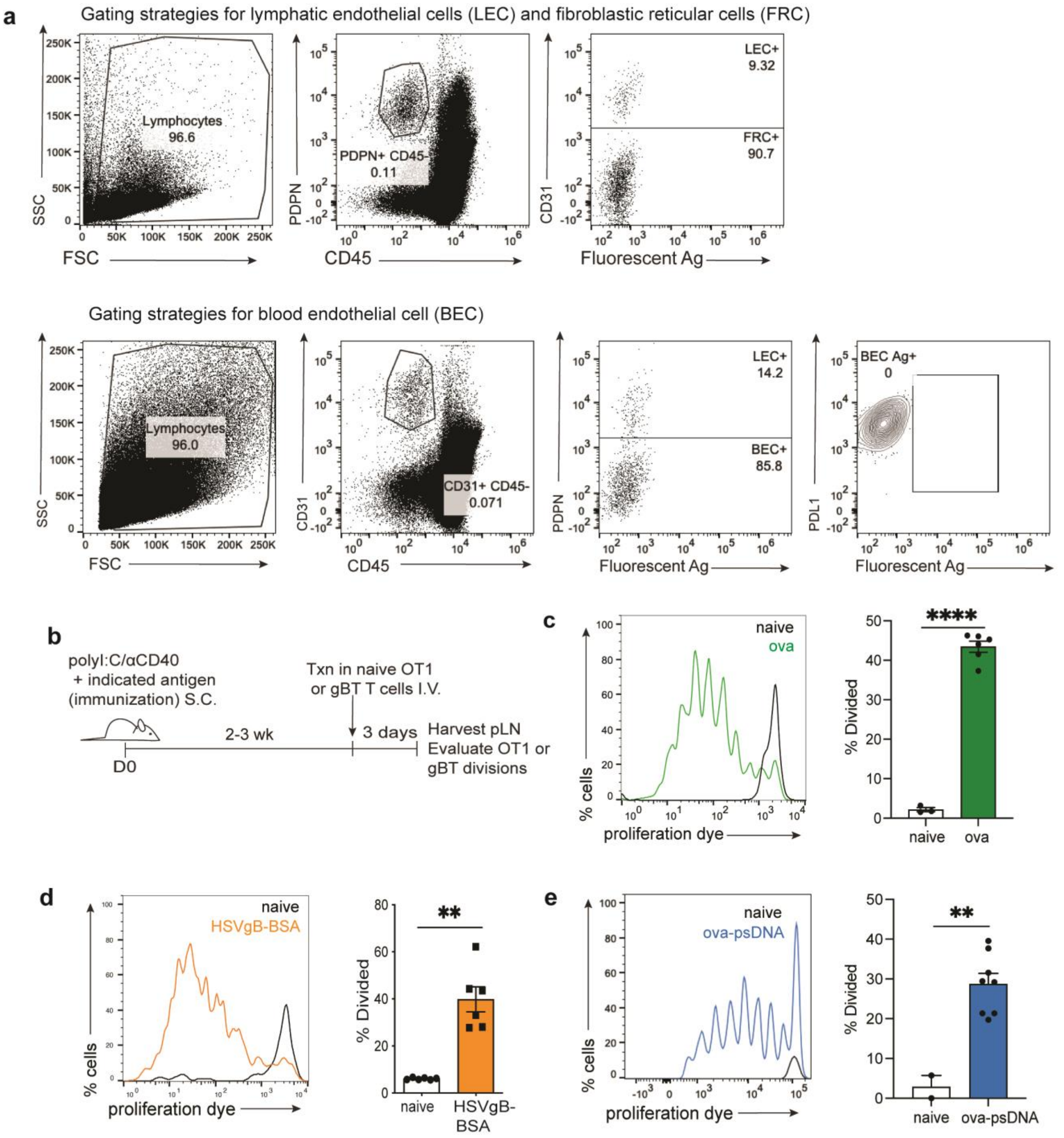

Supplementary Figure 1 - (A) Gating strategies for lymphatic endothelial cells (LEC), fibroblastic reticular cells (FRC) and blood endothelial cells (BEC). Lymph node stromal cells from the draining lymph node of euthanized mice were gated as CD45-. LECs were gated as CD31+ and PDPN+, BECs were gated as CD31+ and PDPN- and FRCs were gated as CD31- and PDPN+. (B) Experimental schematic for Supplementary Figure 1 C-E. Mice were immunized with indicated fluorescent antigens with polyI:C and αCD40. After 2-3 weeks, CFSE or VPD-labeled naïve OT1 or gBT were transferred intravenously into mice and 3 days later OT1 and gBT divisions were assessed. (C) Representative histograms of ova and quantification of percent divided of OT1 T cells. The percent divided was quantitated as described in the supplementary methods section. (D) Same as C, except with HSVgB-BSA and gBT T cells. (E) Same as C, except with ova-psDNA. Statistical analysis was done using an unpaired t-test where the p-value of naïve and immunized mice is <0.0001. Errors bars are mean ± standard error of the mean. In each experiment, at least n=3 mice per group were evaluated and the experiment was repeated n=2 times. Shown is the combined data from both experiments.

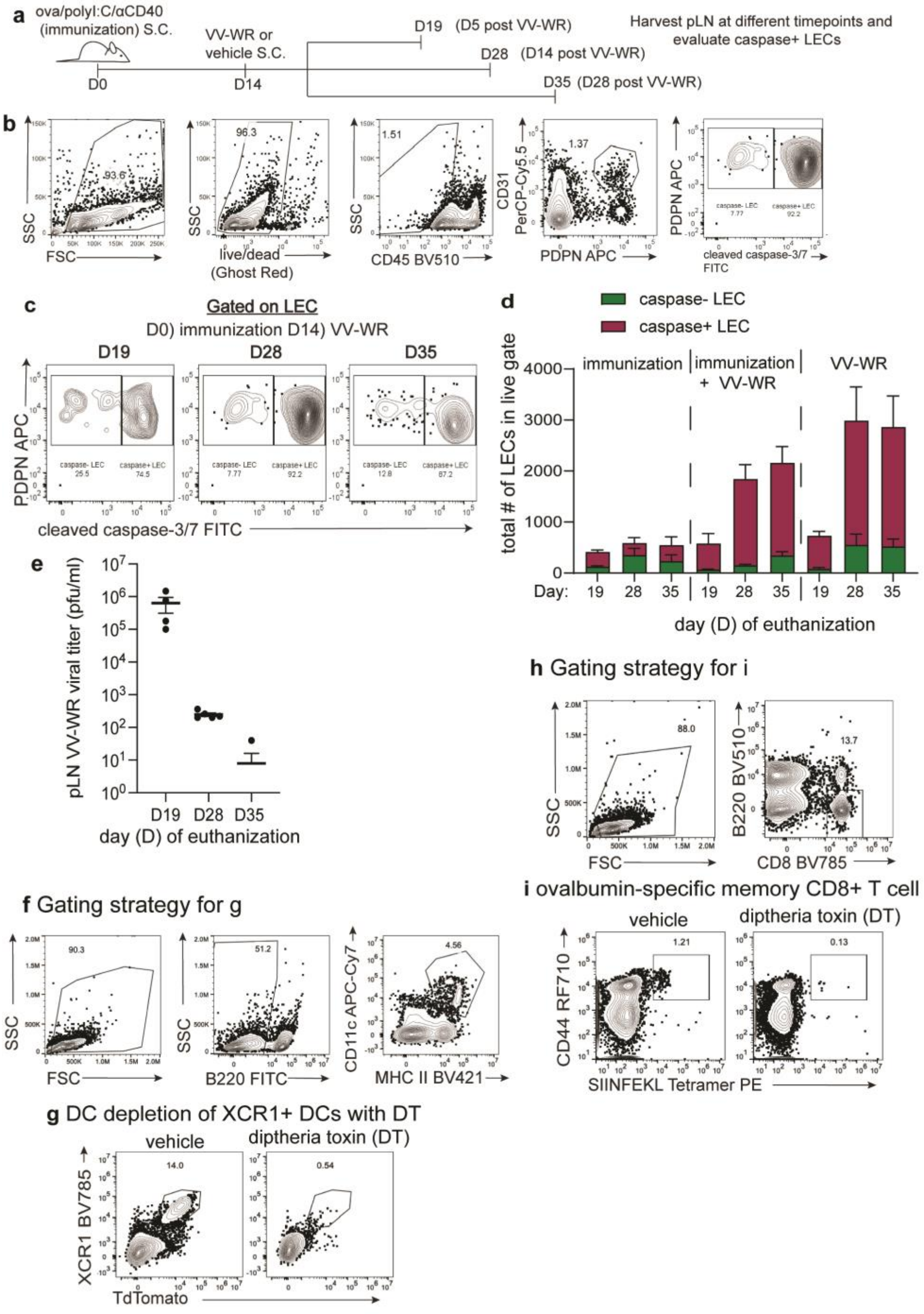

Supplementary Figure 2 - (A) Experimental schematic for B-F. Mice were immunized and infected with VV-WR at indicated time points. Popliteal LN were harvested at respective time points and cleaved caspase+ LECs were evaluated. (B) Gating strategies for gating on live LEC and cleaved caspase+ LEC. (C) Representative flow plots of LEC positive for cleaved caspase-3/7+ at each respective time point. (D) Quantification of the total number of LEC in live gate that expressed caspase or not for each respective treatment. Repeated 2-3 times with 2-5 mice per group, shown is data from 1 experiment with 5 mice per group. (E) Viral titer (pfu/mL) of mice infected with VV-WR (no subunit vaccine) at indicated timepoint. (F) Gating strategy for DCs in G. (G) Example flow plots for paper figure 2C. (H) Gating strategy for CD8 T cells in I. (I) Example flow plots for paper figure 2D. Statistical analysis was done using an unpaired t-test where the p-value of each comparison is <0.0001. Errors bars are mean ± standard error of the mean. In each experiment, at least n=3 mice per group were evaluated and the experiment was repeated n=2 times. Shown is the combined data from both experiments. For viral plaque assay, n=5 per timepoint (at D35 n=4 are 0 pfu/mL).

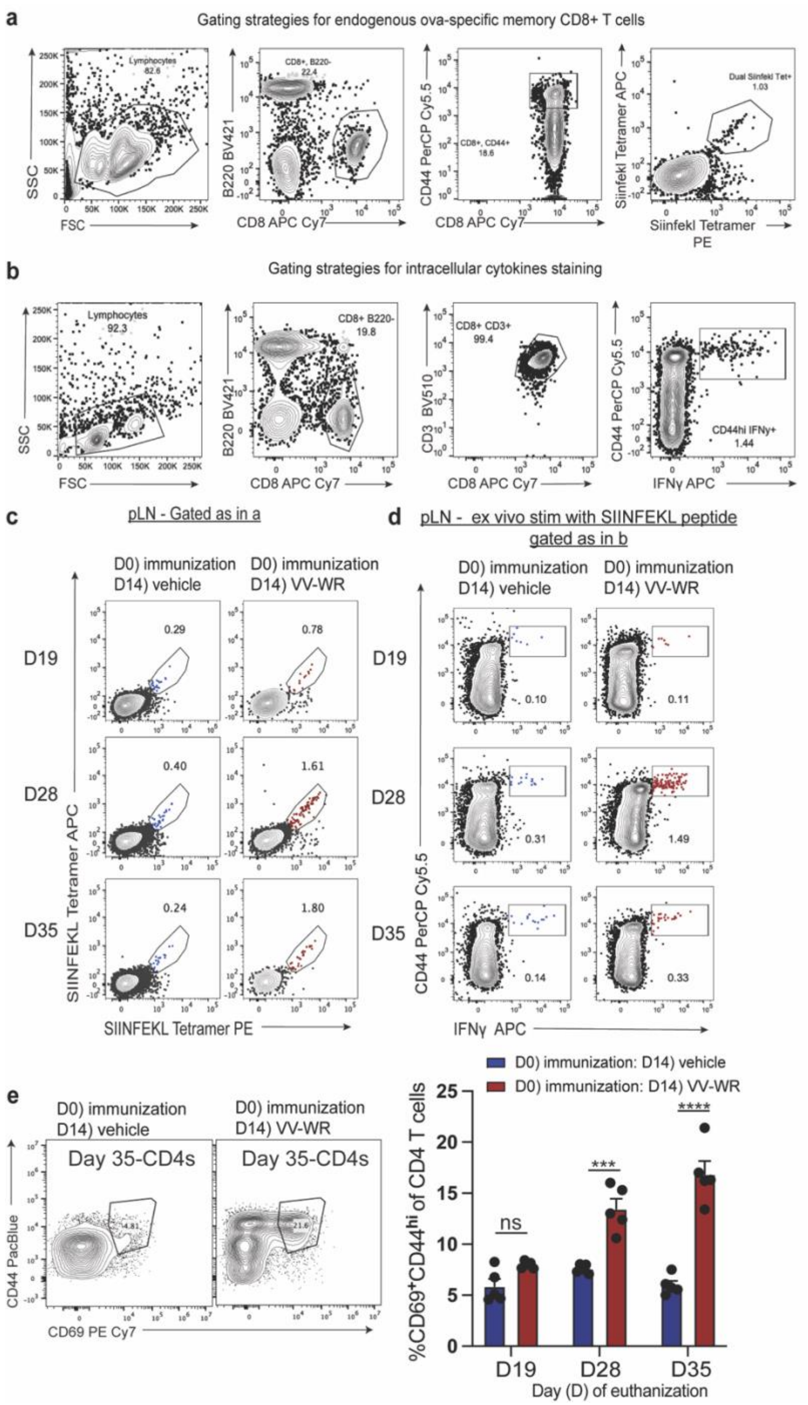

Supplementary Figure 3 - (A) Gating strategies for endogenous ova-specific memory CD8+ T cells. SIINFEKL tetramer staining with two different fluorescent tags was used to confirm specificity. (B) Gating strategies for intracellular cytokine stain. Cells were gated on B220-/CD8+. *IFNγ*-producing CD44^{hi} CD8+ T cells were assessed. (C) Representative flow cytometric plots of endogenous ova-specific H2-Kb SIINFEKL tetramer+ CD8+ T cells were evaluated using SIINFEKL-tetramer PE and SIINFEKL-tetramer APC. Prior to tetramer, cells were gated as B220-/CD8+/CD44^{hi}. Blue represents mice that were injected subcutaneously with vehicle at D14 and red represents mice that were infected with VV-WR at D14. (D) Cells at respective time points were stimulated *ex vivo* with SIINFEKL peptide for 4-6 hrs to evaluate cytokine production. Respective flow cytometric plots show IFNγ production of B220-/CD8+/CD44^{hi} cells. (E) Example flow plots for CD4 T cells gated as either CD4+ B220-, CD8- or CD8-, CD19- lymphocytes and quantification of percent of CD69+CD44+ of parent gate at indicated days from schematic outlined in Figure 3A. Blue bars represent mice injected with Vehicle at Day 14 and red bars represent mice injected with VV-WR at Day 14. Errors bars are mean ± standard error of the mean. ***$p<0.001$, ****$p<0.0001$. Experiment was repeated at least twice with n=2-3 mice per group. Combined data is shown.

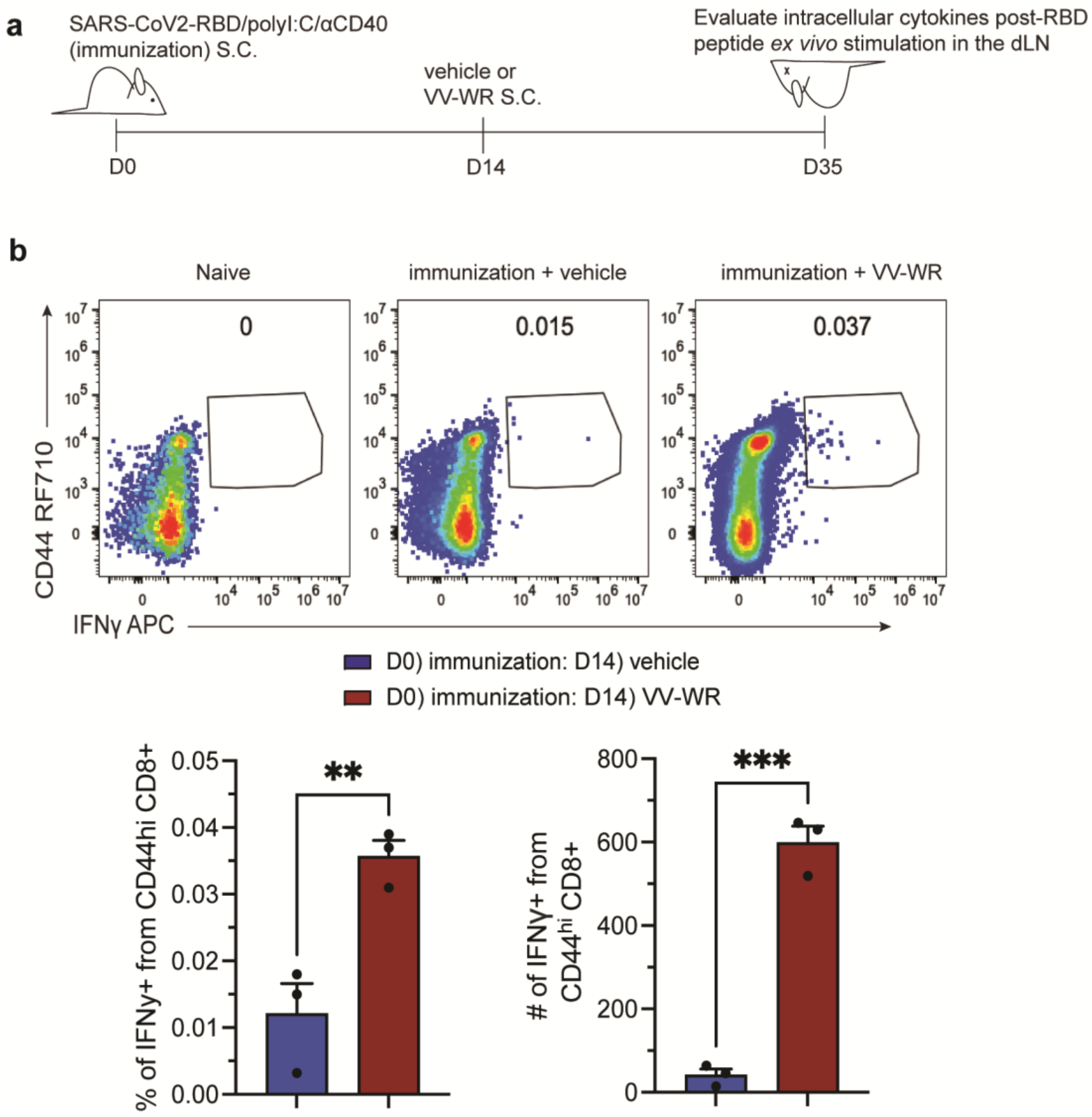

Supplementary Figure 4 – (A) Experimental design for B. (B) Example flow plots from indicated groups showing CD44 positive IFNγ positive cells after immunization with or without VV-WR. Quantification of percent and number of cells shown at Day 35 when mice were euthanized. Blue bars represent mice injected with vehicle at Day 14 and red bars represent mice injected with VV-WR at Day 14. Errors bars are mean ± standard error of the mean. Experiment was repeated with similar results and n=3 mice per group. $^{**}p<0.01$, $^{***}p<0.001$

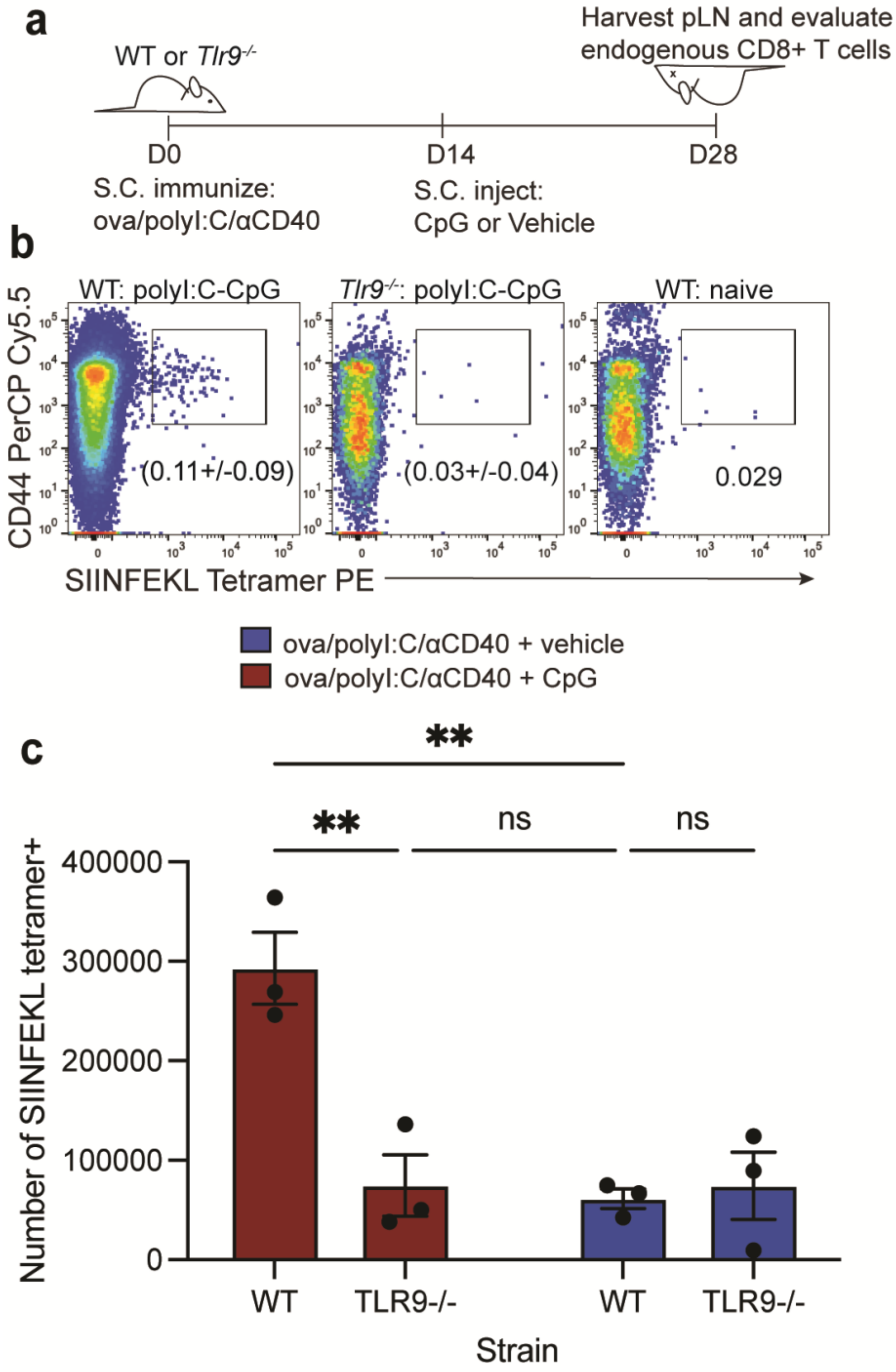

Supplementary Figure 5 - (A) Experimental schematic for B-C. WT or *Tlr9-/-* mice were immunized with the ova/polyI:C/αCD40 vaccine and then injected with CpG alone after 2 weeks. Popliteal LN were harvested and endogenous ova-specific memory CD8+ T cells were evaluated 2 weeks post-CpG. (D) Representative flow plots of WT or *Tlr9-/-* mice receiving secondary CpG or vehicle. (E) Quantification of the total number of endogenous ova-specific memory CD8+ T cells. Statistical analysis was done using an unpaired t-test where the p-value of the immunized mice that were secondarily given CpG or not is <0.0001. Errors bars are mean ± standard error of the mean. In each experiment, at least n=3 mice per group were evaluated and the experiment was repeated n=2 times with similar results. Shown is the representative data from one of the experiments.

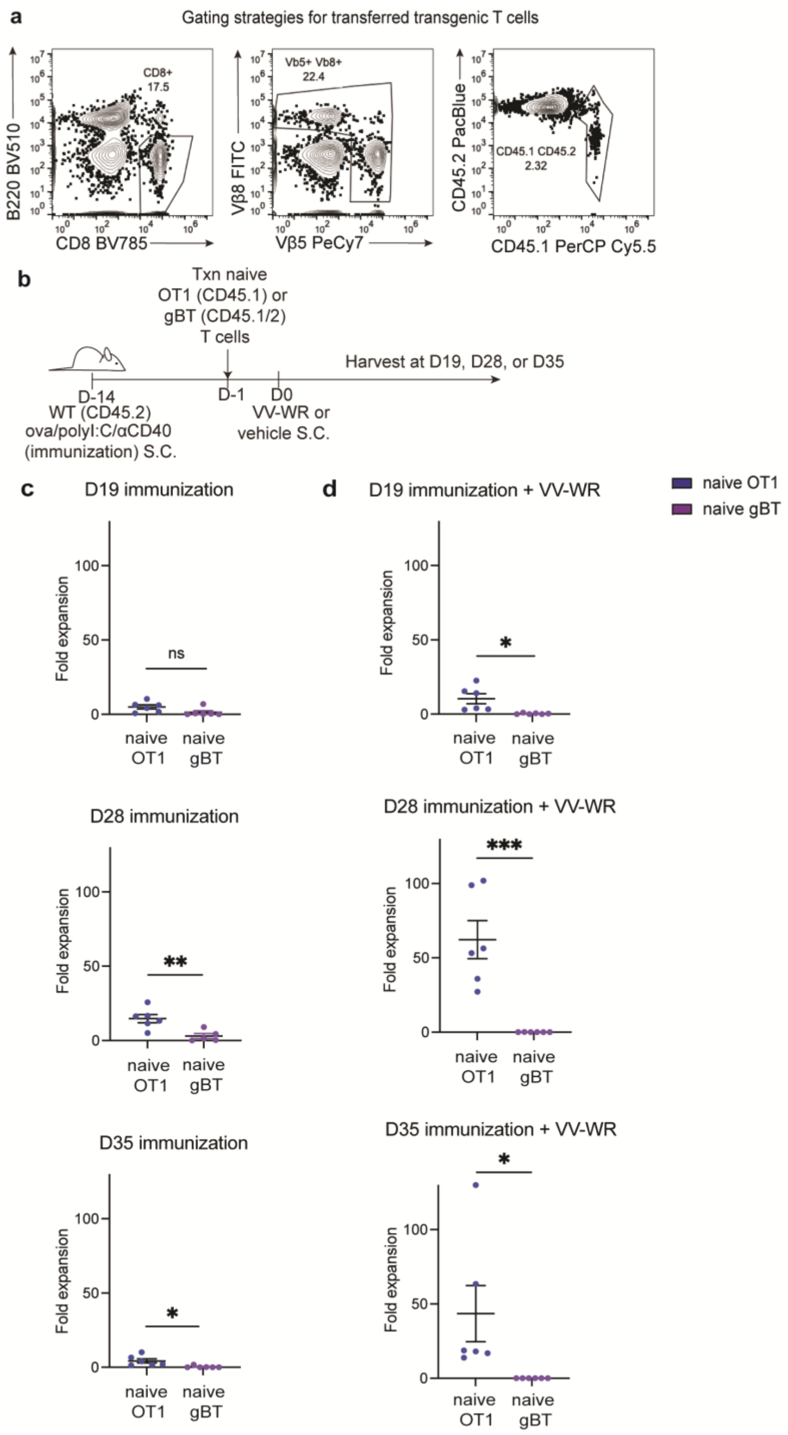

Supplementary Figure 6 - (A) Gating strategies for transferred TCR transgenic T cells. Cells were gated on CD8+/B220- followed by Vβ8 (gBT) and Vβ5 (OT1) for TCR transgenic T cells. The cells were then separated from the endogenous T cells via different congenic markers (e.g. OT1 – CD45.1/1, gBT – CD45.1/2 into CD4.2.2 host or OT1-CD45.1/2, gBT – CD45.2/2 into CD45.1/1 host). (B) Mice were immunized and infected with VV-WR as Figure 3. One day prior to VV-WR infection, naïve OT1 and naïve gBT were transferred into antigen-bearing WT host and naïve host. Popliteal LN were harvested at indicated time points post-VV-WR. (C,D) The fold expansion was calculated as the total number of naïve OT1 or naive gBT in antigen-bearing mice over the total number of naive OT1 or naive gBT in the naïve WT host at each respective time point. Statistical analysis was done using an unpaired t-test where the p-value of the naïve OT1 and naïve gBT is <0.0001. Errors bars are mean ± standard error of the mean. In each experiment, at least n=3 mice per group were evaluated and the experiment was repeated n=2 times. Shown in Supplementary Figure 5C-D is the combined data from both experiments.

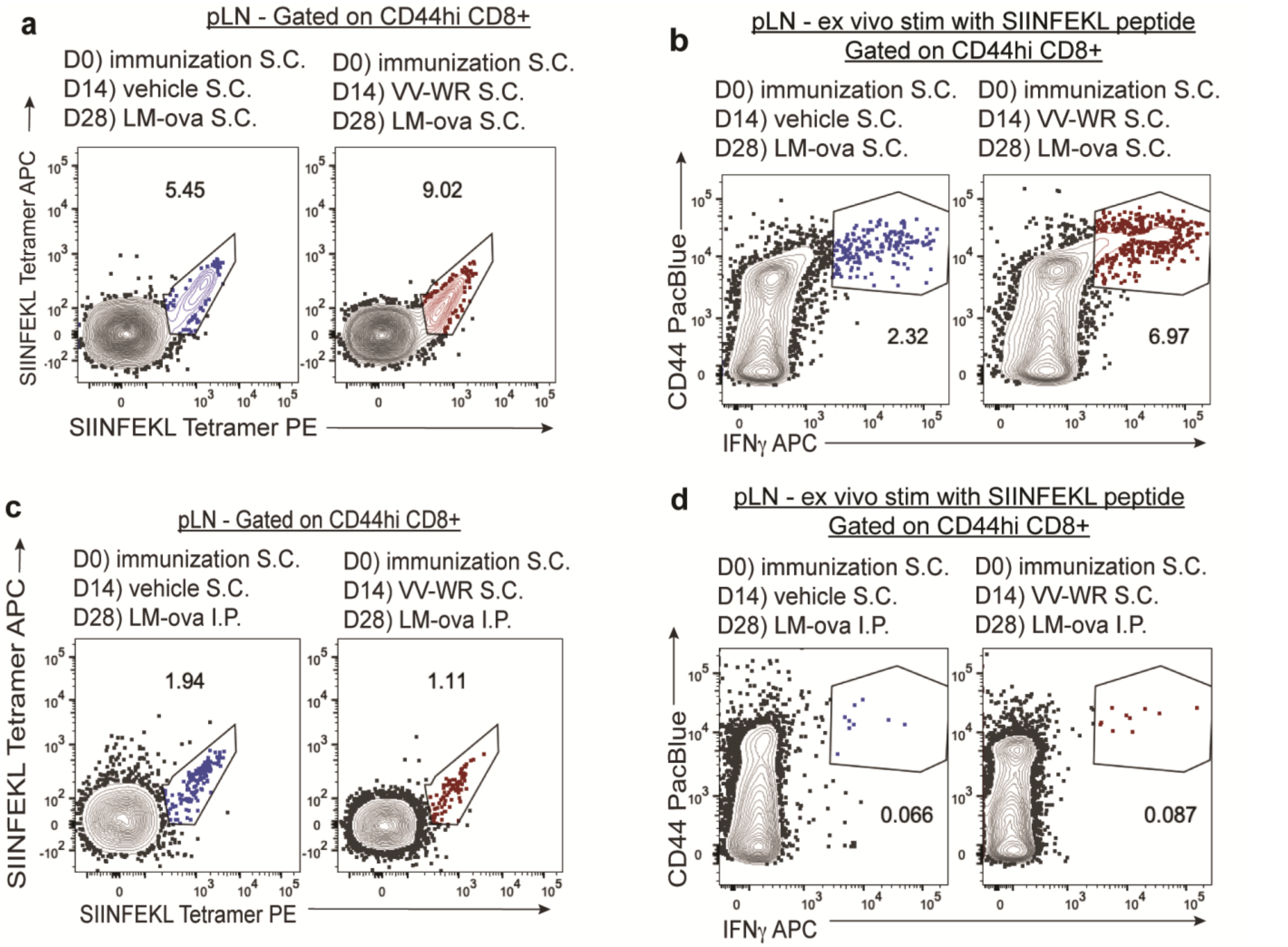

Supplementary Figure 7. (A) Representative flow plots of mice given LM-ova subcutaneously (S.C). Blue represents mice that were injected subcutaneously with vehicle at D14 and red represents mice that were infected with VV-WR at D14. Cells were evaluated using SIINFEKL-tetramer PE and SIINFEKL-tetramer APC. Previous gates were B220-/CD8+/CD44^{hi}. (B) Representative flow plots of draining lymph node cells following *ex vivo* stimulation with SIINFEKL peptide for 4-6 hrs. Respective flow cytometric plots show IFNγ production of cells gated previously on B220-/CD8+/CD44^{hi}. (C) Same as A except LM-ova was administered intraperitoneally (I.P.) (D) Same as B except LM-ova was administered intraperitoneally.

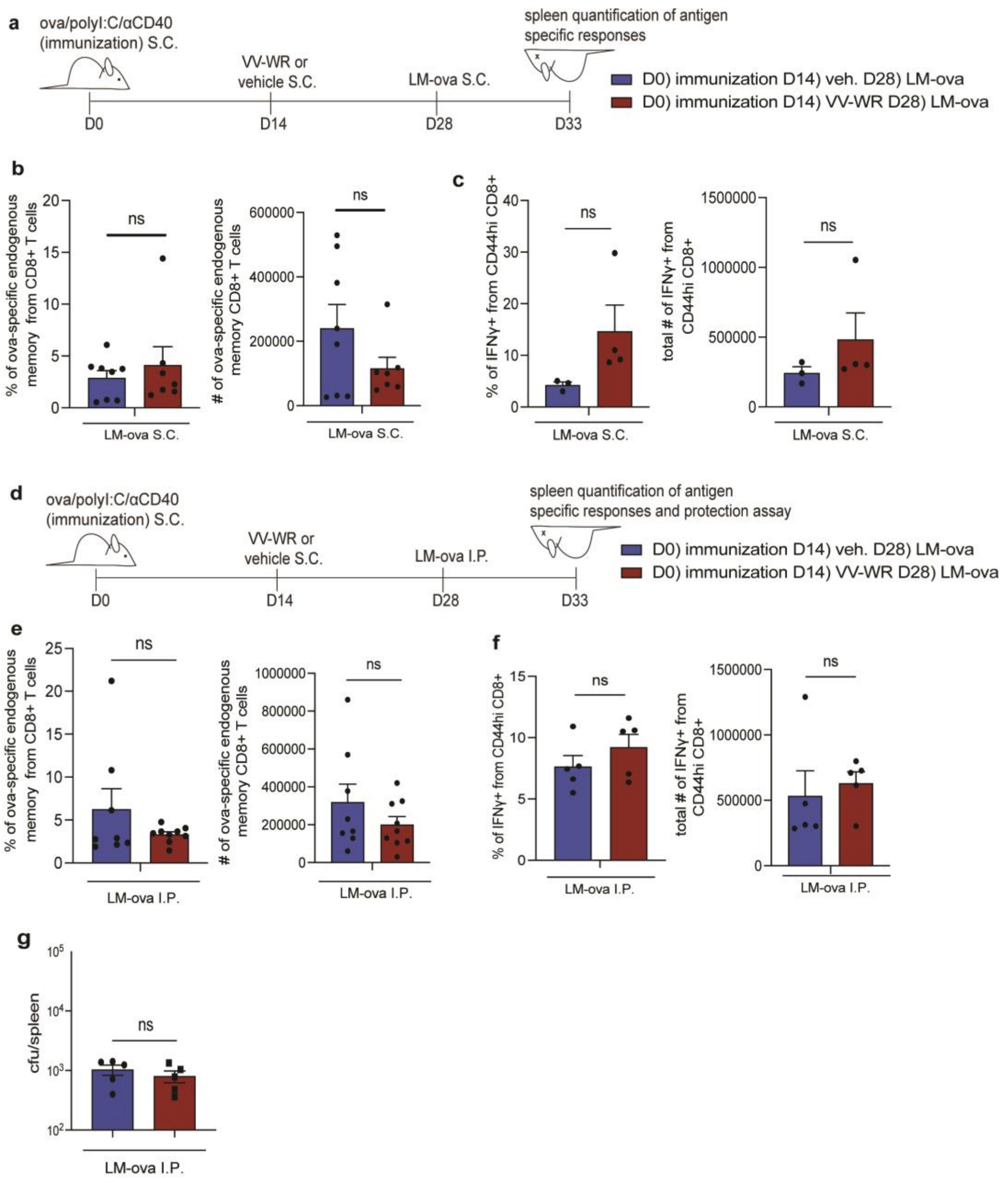

Supplementary Figure 8– (A) Experimental schematic for B-C. Mice were immunized with ova/polyI:C/αCD40 and infected with VV-WR 2 weeks later. Mice were then challenged LM-ova subcutaneously (S.C.) Five days post-LM-ova, the spleens were harvested and processed (B) Cells were evaluated using SIINFEKL-tetramer PE and SIINFEKL-tetramer APC. The previous gates were B220-/CD8+/CD44^{hi}. Quantification of frequency and number of ova-specific endogenous memory CD8+ T cells in the spleen 5 days post-LM-ova S.C. Blue represents mice that were injected with vehicle D14, while red represents mice were infected with VV-WR at D14. (C) The cells from the spleen were stimulated *ex vivo* with SIINFEKL peptide for 4-6 hrs to evaluate cytokine production. Quantification of frequency and number of IFNγ-producing CD44^{hi} CD8+T cells in the spleen. (D) Experimental schematic for E-G. Mice were challenged with LM-ova intraperitoneally (I.P.) (E) Same as B, except for the mice were challenged with LM-ova I.P. (F), Same as C, except for the mice were challenged with LM-ova I.P. (G) Quantification from colonies-forming units (CFU). Spleens from LM-ova-infected I.P. was processed as described in the methods section. Homogenized tissues were plated on BHI + erythromycin plates and colonies were counted after 3 days. Statistical analysis was done using an unpaired t-test where the p-value between vaccine + vehicle + LM-ova (blue bar) and vaccine + VV-WR + LM-ova (red bar) is <0.0001. Errors bars are mean ± standard error of the mean. In each experiment, n=3-5 mice per group were evaluated and the experiment was repeated n=2 times. Shown is the combined data from both experiments.

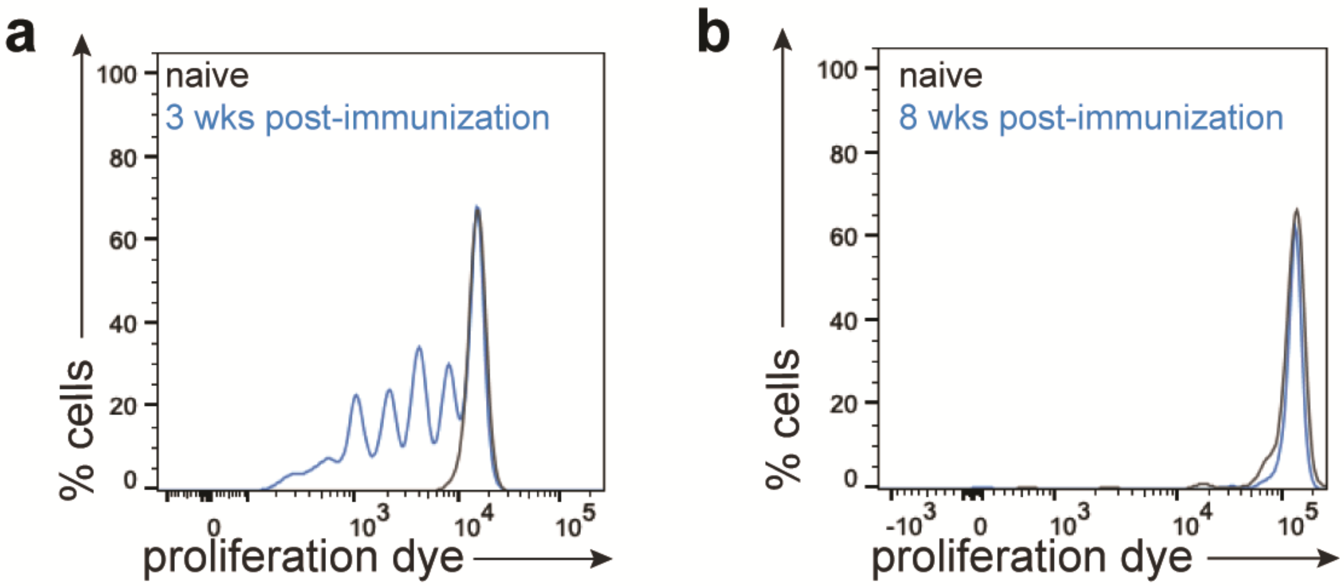

Supplementary Figure 9 - (A) Mice were immunized with ovalbumin/polyI:C/αCD40. 3 weeks post-immunization, VPD labeled-naïve OT1 T cells were transferred into immunized mice. 3 days later, popliteal LN were harvested and OT1 division was assessed for residual archived antigens. (B) Same as A, except naïve OT1 T cells were transferred in at 8 weeks post-immunization.
