# Supplementary material for: Immunization-induced antigen archiving enhances local memory CD8+ T cell responses following an unrelated viral infection
Source: NPJ Vaccines. 2024 Mar 21;9:66. doi: 10.1038/s41541-024-00856-6 (PMC10957963; doi:10.1038/s41541-024-00856-6)
Supplement: Supplementary file 2 — REPORTING SUMMARY [file 41541_2024_856_MOESM2_ESM.pdf]

nature portfolio

Corresponding author(s): Dr Beth Tamburini

Last updated by author(s): Feb 18, 2024

Reporting Summary

Nature Portfolio wishes to improve the reproducibility of the work that we publish. This form provides structure for consistency and transparency in reporting. For further information on Nature Portfolio policies, see our Editorial Policies and the Editorial Policy Checklist.

Statistics

For all statistical analyses, confirm that the following items are present in the figure legend, table legend, main text, or Methods section.

n/a	Confirmed	
☐	☒	The exact sample size (*n*) for each experimental group/condition, given as a discrete number and unit of measurement
☐	☒	A statement on whether measurements were taken from distinct samples or whether the same sample was measured repeatedly
☐	☒	The statistical test(s) used AND whether they are one- or two-sided<br>*Only common tests should be described solely by name; describe more complex techniques in the Methods section.*
☐	☒	A description of all covariates tested
☐	☒	A description of any assumptions or corrections, such as tests of normality and adjustment for multiple comparisons
☐	☒	A full description of the statistical parameters including central tendency (e.g. means) or other basic estimates (e.g. regression coefficient) AND variation (e.g. standard deviation) or associated estimates of uncertainty (e.g. confidence intervals)
☐	☒	For null hypothesis testing, the test statistic (e.g. *F*, *t*, *r*) with confidence intervals, effect sizes, degrees of freedom and *P* value noted<br>*Give P values as exact values whenever suitable.*
☒	☐	For Bayesian analysis, information on the choice of priors and Markov chain Monte Carlo settings
☒	☐	For hierarchical and complex designs, identification of the appropriate level for tests and full reporting of outcomes
☒	☐	Estimates of effect sizes (e.g. Cohen's *d*, Pearson's *r*), indicating how they were calculated

Our web collection on statistics for biologists contains articles on many of the points above.

Software and code

Policy information about availability of computer code

Data collection	N/A
Data analysis	N/A

For manuscripts utilizing custom algorithms or software that are central to the research but not yet described in published literature, software must be made available to editors and reviewers. We strongly encourage code deposition in a community repository (e.g. GitHub). See the Nature Portfolio guidelines for submitting code & software for further information.

Data

Policy information about availability of data

All manuscripts must include a data availability statement. This statement should provide the following information, where applicable:

- Accession codes, unique identifiers, or web links for publicly available datasets
- A description of any restrictions on data availability
- For clinical datasets or third party data, please ensure that the statement adheres to our policy

All data and material within this article will be available upon reasonable request to the corresponding author.

Research involving human participants, their data, or biological material

Policy information about studies with human participants or human data. See also policy information about sex, gender (identity/presentation), and sexual orientation and race, ethnicity and racism.

Reporting on sex and gender	N/A
Reporting on race, ethnicity, or other socially relevant groupings	N/A
Population characteristics	N/A
Recruitment	N/A
Ethics oversight	N/A

Note that full information on the approval of the study protocol must also be provided in the manuscript.

Field-specific reporting

Please select the one below that is the best fit for your research. If you are not sure, read the appropriate sections before making your selection.

☒ Life sciences ☐ Behavioural & social sciences ☐ Ecological, evolutionary & environmental sciences

For a reference copy of the document with all sections, see nature.com/documents/nr-reporting-summary-flat.pdf

Life sciences study design

All studies must disclose on these points even when the disclosure is negative.

Sample size	Each in vivo analysis was performed with 3-5 mice per group as determined by a power calculation using the assumption (based on prior data) that there will be at least a two fold change with a standard deviation of less than 0.5. To calculate numbers we performed a power calculation with an alpha of 0.5 and a 1-beta of 0.80 to determine at least 3 mice per group should be evaluated. Exact replicates and numbers are provided in the figure legends.
Data exclusions	Data was only excluded based on specific criteria. In the XCR1 DTR depletion study one mouse did not demonstrate a depletion in XCR1+ DCs therefore we excluded this mouse from the analysis.
Replication	All experiments were repeated with similar results demonstrating the reproducibility. In most cases all replicates are shown. In some cases time-points were repeated separately from the time-course and therefore not included in the figure. However, in each case the experimental samples were different from the control sample values as demonstrated in the figure.
Randomization	Wild-type or transgenic mice were included randomly into each group. Collection and analysis of data was done exactly the same for each group.
Blinding	Data collection was blinded until analysis.

Reporting for specific materials, systems and methods

We require information from authors about some types of materials, experimental systems and methods used in many studies. Here, indicate whether each material, system or method listed is relevant to your study. If you are not sure if a list item applies to your research, read the appropriate section before selecting a response.

Materials & experimental systems

n/a	Involved in the study	
☐	☒	Antibodies
☐	☐	Eukaryotic cell lines
☐	☐	Palaeontology and archaeology
☐	☒	Animals and other organisms
☐	☐	Clinical data
☐	☐	Dual use research of concern
☐	☐	Plants

Methods

n/a	Involved in the study	
☐	☐	ChIP-seq
☐	☒	Flow cytometry
☐	☐	MRI-based neuroimaging

Antibodies

Antibodies used	Anti-mouse CD40 (Rat monoclonal) BioXcell FGK4.5;Anti-mouse CD8 (Rat monoclonal) Biolegend 53-6.7;Anti-mouse/human B220/ CD45R (Rat monoclonal) Biolegend RA3-6B2;Anti-mouse CD3 (Rat monoclonal) Biolegend 17A2;Anti-mouse CD4 Biolegend RM4-5;Anti-mouse CD69 Biolegend H1.2F3;Anti-mouse CD44 (Rat monoclonal) Biolegend IM7;Anti-mouse CD19 Biolegend 6D5;IFN Biolegend XMG1.2;Vb5 Biolegend MR9-4;Vb8 Biolegend KJ16-133.18;Anti-mouse CD45.1 (Mouse monoclonal) Biolegend A-20;Anti-mouse CD45.2 (Mouse monoclonal) Biolegend 104;Anti-mouse CD45 (Rat monoclonal) Biolegend 30-F11;Anti-mouse CD31 Biolegend 390;Anti-mouse PD-L1 (Rat monoclonal) Biolegend 10F.9G2;Anti-mouse podoplanin/gp38 Biolegend 8.1.1
Validation	Each antibody is quality control tested by immunofluorescent staining with flow cytometric analysis and each antibody is purified by affinity chromatography and conjugated with a fluorophor. In addition negative controls are used for staining to validate antibodies used by including a fluorescence minus one control or isotype control to validate the positive staining of the antibody. For the FGK4.5 antibody from BioXcell for use in vivo the antibody is <2EU/mg by LAL gel clotting assay and binding validation is performed to confirm specific binding to purfied CD40.

Eukaryotic cell lines

Policy information about cell lines and Sex and Gender in Research

Cell line source(s)	N/A
Authentication	N/A
Mycoplasma contamination	N/A
Commonly misidentified lines (See ICLAC register)	N/A

Palaeontology and Archaeology

Specimen provenance	N/A
Specimen deposition	N/A
Dating methods	N/A

☐ Tick this box to confirm that the raw and calibrated dates are available in the paper or in Supplementary Information.

Ethics oversight	N/A

Note that full information on the approval of the study protocol must also be provided in the manuscript.

Animals and other research organisms

Policy information about studies involving animals; ARRIVE guidelines recommended for reporting animal research, and Sex and Gender in Research

Laboratory animals	mus musculus, C57BL/6 age 5-10 weeks
Wild animals	N/A
Reporting on sex	Sex was not determined to be a variable in this study. Both male and female mice were used initially and no differences were observed so the majority of the mice used in this study were female.
Field-collected samples	N/A
Ethics oversight	These studies were approved by the Institutional Animal Care and Use Committee (IACUC) at the University of Colorado Anschutz Medical Campus.

Note that full information on the approval of the study protocol must also be provided in the manuscript.

Clinical data

Policy information about clinical studies

All manuscripts should comply with the ICMJE guidelines for publication of clinical research and a completed CONSORT checklist must be included with all submissions.

Clinical trial registration	N/A
Study protocol	N/A
Data collection	N/A
Outcomes	N/A

Dual use research of concern

Policy information about dual use research of concern

Hazards

Could the accidental, deliberate or reckless misuse of agents or technologies generated in the work, or the application of information presented in the manuscript, pose a threat to:

No	Yes	
☒	☐	Public health
☒	☐	National security
☒	☐	Crops and/or livestock
☒	☐	Ecosystems
☒	☐	Any other significant area

Experiments of concern

Does the work involve any of these experiments of concern:

No	Yes	
☒	☐	Demonstrate how to render a vaccine ineffective
☒	☐	Confer resistance to therapeutically useful antibiotics or antiviral agents
☒	☐	Enhance the virulence of a pathogen or render a nonpathogen virulent
☒	☐	Increase transmissibility of a pathogen
☒	☐	Alter the host range of a pathogen
☒	☐	Enable evasion of diagnostic/detection modalities
☒	☐	Enable the weaponization of a biological agent or toxin
☒	☐	Any other potentially harmful combination of experiments and agents

Plants

Seed stocks	N/A
Novel plant genotypes	N/A
Authentication	N/A

ChIP-seq

Data deposition

☐ Confirm that both raw and final processed data have been deposited in a public database such as GEO.

☐ Confirm that you have deposited or provided access to graph files (e.g. BED files) for the called peaks.

Data access links<br>*May remain private before publication.*	NA
Files in database submission	NA
Genome browser session<br>(e.g. UCSC)	NA

Methodology

Replicates	NA
Sequencing depth	NA
Antibodies	NA
Peak calling parameters	NA
Data quality	NA
Software	NA

Flow Cytometry

Plots

Confirm that:

☒ The axis labels state the marker and fluorochrome used (e.g. CD4-FITC).

☒ The axis scales are clearly visible. Include numbers along axes only for bottom left plot of group (a 'group' is an analysis of identical markers).

☒ All plots are contour plots with outliers or pseudocolor plots.

☒ A numerical value for number of cells or percentage (with statistics) is provided.

Methodology

Sample preparation	Draining LNs and spleens were harvested and processed by frosted glass slide maceration. Red blood cells from the spleens were lysed using Ammonium-Chloride-Potassium (ACK) lysis buffer. The cells were filtered, washed, and suspended in complete RPMI with 2.5% fetal bovine serum (FBS). Cells were stained with anti-mouse CD8 antibody (clone: 53-6.7) and both SIINFEKL tetramer-PE and SIINFEKL tetramer-APC (NIH tetramer core facility) for 1 h at 37�C. Cells were then stained for additional surface markers (CD3, CD4, CD69, CD44, B220, KLRG1, CD127-see table for clone numbers) for 30 min at 37�C. After washing, samples were run on BD Canto II flow cytometer or Beckman Coulter Cytoflex LX flow cytometer. For intracellular cytokine staining, single-cell suspensions were ex vivo stimulated in brefeldin A (1 �g/ml)) with or without (2��g/ml) SIINFEKL peptide for 4-6 h at 37�C. After stimulation, cells were stained with anti-CD8, -B220, -CD3, and -CD44 antibodies (see table). Cells were then fixed with 1% paraformaldehyde and 3% sucrose for 10 min in the dark at room temperature. Cells were washed twice with FACS buffer (0.1% bovine serum albumin (BSA), 1x Hank's buffered saline solution, 2 mM ethylene diamine tetra acetic acid (EDTA) and 0.02% sodium azide) and then permeabilized with 1x perm wash (BD Cat. No. 554723). The cells were then stained for IFN� (clone: XMG1.2) in 1x perm wash. The following day, the cells were washed in perm buffer 2 times and resuspended in FACS buffer before acquiring
Instrument	BD-Canto II or Beckman Coulter CytoFlex-LX N0-V5-B3-Y5-R3-I0
Software	All flow cytometry data were analyzed with FlowJo software and statistical analysis and graphing was done using Graphpad Prism software.
Cell population abundance	N/A

Gating strategy

For lymph node stromal cells: Cells were gated by forward scatter and side scatter; CD45XPDPN;CD31Xfluorescent antigen;PD-L1Xfluorescent antigen
For CD8 T cells: Forward scatter X side scatter; B220XCD8;CD3XCD8;CD44Xtetramer or CD8;tetramerXtetramer
For DCs:forward scatter X side scatter; B220-;CD11cXMHCII; XCR1Xtdtomato

☒ Tick this box to confirm that a figure exemplifying the gating strategy is provided in the Supplementary Information.

Magnetic resonance imaging

Experimental design

Design type	na
Design specifications	na
Behavioral performance measures	na

Acquisition

Imaging type(s)	na
Field strength	na
Sequence & imaging parameters	na
Area of acquisition	na

Diffusion MRI ☐ Used ☐ Not used

Preprocessing

Preprocessing software	na
Normalization	na
Normalization template	na
Noise and artifact removal	na
Volume censoring	na

Statistical modeling & inference

Model type and settings	na
Effect(s) tested	na

Specify type of analysis: ☐ Whole brain ☐ ROI-based ☐ Both

Statistic type for inference (See Eklund et al. 2016)	na
Correction	na

Models & analysis

n/a	Involved in the study
☐	☐ Functional and/or effective connectivity
☐	☐ Graph analysis
☐	☐ Multivariate modeling or predictive analysis

Functional and/or effective connectivity	na
Graph analysis	na
Multivariate modeling and predictive analysis	na
